# Characterization of an autonomous pathway complex that promotes flowering in *Arabidopsis*

**DOI:** 10.1093/nar/gkac551

**Published:** 2022-06-29

**Authors:** Pei-Lin Qi, Hao-Ran Zhou, Qiang-Qiang Zhao, Chao Feng, Yong-Qiang Ning, Yin-Na Su, Xue-Wei Cai, Dan-Yang Yuan, Zhao-Chen Zhang, Xiao-Min Su, Shan-Shan Chen, Lin Li, She Chen, Xin-Jian He

**Affiliations:** National Institute of Biological Sciences, Beijing 102206, China; PTN Joint Graduate Program, School of Life Sciences, Tsinghua University, Beijing 100084, China; National Institute of Biological Sciences, Beijing 102206, China; National Institute of Biological Sciences, Beijing 102206, China; Graduate School of Peking Union Medical College, Beijing 100730, China; National Institute of Biological Sciences, Beijing 102206, China; National Institute of Biological Sciences, Beijing 102206, China; National Institute of Biological Sciences, Beijing 102206, China; National Institute of Biological Sciences, Beijing 102206, China; National Institute of Biological Sciences, Beijing 102206, China; National Institute of Biological Sciences, Beijing 102206, China; National Institute of Biological Sciences, Beijing 102206, China; National Institute of Biological Sciences, Beijing 102206, China; National Institute of Biological Sciences, Beijing 102206, China; National Institute of Biological Sciences, Beijing 102206, China; Tsinghua Institute of Multidisciplinary Biomedical Research, Tsinghua University, Beijing 100084, China; National Institute of Biological Sciences, Beijing 102206, China; Tsinghua Institute of Multidisciplinary Biomedical Research, Tsinghua University, Beijing 100084, China

## Abstract

Although previous studies have identified several autonomous pathway components that are required for the promotion of flowering, little is known about how these components cooperate. Here, we identified an autonomous pathway complex (AuPC) containing both known components (FLD, LD and SDG26) and previously unknown components (EFL2, EFL4 and APRF1). Loss-of-function mutations of all of these components result in increased *FLC* expression and delayed flowering. The delayed-flowering phenotype is independent of photoperiod and can be overcome by vernalization, confirming that the complex specifically functions in the autonomous pathway. Chromatin immunoprecipitation combined with sequencing indicated that, in the AuPC mutants, the histone modifications (H3Ac, H3K4me3 and H3K36me3) associated with transcriptional activation are increased, and the histone modification (H3K27me3) associated with transcriptional repression is reduced, suggesting that the AuPC suppresses *FLC* expression at least partially by regulating these histone modifications. Moreover, we found that the AuPC component SDG26 associates with *FLC* chromatin via a previously uncharacterized DNA-binding domain and regulates *FLC* expression and flowering time independently of its histone methyltransferase activity. Together, these results provide a framework for understanding the molecular mechanism by which the autonomous pathway regulates flowering time.

## INTRODUCTION

The proper timing of the transition from vegetative to reproductive growth is crucial for the reproductive success of plants under diverse environmental conditions. In *Arabidopsis thaliana*, the flowering time can be promoted by the autonomous, photoperiod, vernalization and gibberellin signaling pathways (1–5). The MADS box transcription factor FLC functions as a key flowering repressor in *Arabidopsis* (6,7). In the winter-annual accessions, the expression of *FLC* is activated by FRI (FRIGIDA), leading to a late-flowering phenotype (8,9). The rapid-flowering accessions such as Col-0 usually harbor a loss-of function mutation in *FRI* and fail to activate the expression of *FLC*, resulting in an early-flowering phenotype (6,7). The autonomous pathway components are required for reducing *FLC* expression in rapid-flowering accessions and in vernalization-treated winter annuals (10–12). Loss-of-function mutations in the autonomous pathway components result in increased *FLC* expression and delayed flowering.

The autonomous pathway components are known to mediate the repression of *FLC* transcription via different mechanisms (13). A group of RNA processing-related autonomous pathway components (FCA, FPA, FLK and FY) are involved in RNA processing and polyadenylation site selection for *COOLAIR*, an antisense long noncoding RNA at the *FLC* locus (10,14–19). FCA and FY facilitate polyadenylation of *COOLAIR* and then promote R-loop resolution, thereby repressing *FLC* transcription (20). Another group of autonomous pathway components including FLD (FLOWERING LOCUS D), LD (LUMINIDEPENDENS) and FVE associate with chromatin and regulate histone modification and transcription (11,12,21,22). FLD is a homolog of the human histone H3K4 demethylase LSD1; LD is a homeodomain-containing protein that functions as a transcriptional repressor of *FLC*; and FVE is a shared subunit of the histone deacetylase complex and the histone H3K27 trimethyltransferase complex (11,12,21–23). FPA and FCA require the histone demethylase FLD to downregulate *FLC* expression (13,19,24), suggesting that *COOLAIR* and histone modifications are likely to cooperatively regulate the *FLC* transcription.

H3K27me3, a histone modification associated with transcriptional repression, is gradually accumulated on *FLC* chromatin during vernalization and is responsible for vernalization-induced flowering in the winter-annual accessions of *Arabidopsis* (25,26). In the rapid-flowering accessions, however, *FLC* chromatin can simultaneously carry both the repressive histone modification H3K27me3 and the active histone modifications H3K4me3 and H3K36me3 even under nonvernalization conditions, thereby forming a bivalent chromatin state at the *FLC* locus (27,28). The conserved histone H3K27 methyltransferase CLF functions as a catalytic subunit of polycomb repressive complex 2 (PRC2) and is responsible for the occurrence of H3K27me3 at *FLC* chromatin in nonvernalized plants (29). Previous studies have shown that depletion of the autonomous pathway component FLD causes an increase in histone acetylation, H3K4me1/2/3 and H3K36me3, and a reduction in H3K27me3 at *FLC* chromatin (11,24,30–36), implicating that the autonomous pathway represses *FLC* expression at least partially by regulating multiple histone modifications. FLD-dependent H3K4me1 demethylation was recently shown to be primarily responsible for repression of *FLC* transcription (35,36). However, H3K4me1 demethylation-independent mechanisms cannot be excluded. Moreover, how different components of the autonomous pathway cooperate to regulate histone modifications is largely unknown.

*FLC* expression is activated by the histone marks H3K4me3 and H3K36me3, which are associated with actively transcribed genes (28,37). The trithorax group (TrxG) histone methyltransferases are responsible for histone methylation on H3K4 and H3K36 sites and thereby mediate transcriptional activation (38–40). The histone H3K4 methyltransferases ATX1 and SDG25/ATXR7 and the histone H3K36 methyltransferase SDG8/EFS can activate *FLC* expression to prevent precocious flowering (39–41). SDG26, a member of the TrxG histone methyltransferase family, has histone methyltransferase activity as determined by an *in vitro* assay (42). Unlike SDG8 that mediates transcriptional activation of *FLC* by promoting H3K36 trimethylation (40), SDG26 functions antagonistically with SDG8 in the regulation of *FLC* expression and flowering time (42,43). SDG26 interacts with the autonomous pathway components LD and FLD and thereby forms a complex that promotes flowering by suppressing *FLC* expression (35). Interestingly, SDG26 was also found to activate the transcription of the critical floral integrator gene *SOC1* by mediating H3K4 trimethylation and H3K36 trimethylation and to thereby promote flowering (44). Thus, further studies are required to determine whether SDG26 regulates flowering by functioning as an autonomous pathway component or by directly promoting *SOC1* expression. Moreover, although SDG26, FLD and LD are known to form a complex, whether the complex contains any previously uncharacterized components and how these components cooperate to regulate flowering time remain elusive.

In this study, we initially performed affinity purification followed by mass spectrometry (AP–MS) to detect proteins co-purified with the autonomous pathway component FLD; we found that FLD interacts not only with the known autonomous pathway components LD and SDG26 but also with three other components. We demonstrate that these components form a multi-subunit autonomous pathway complex (AuPC). In the mutants of these components, *FLC* expression is increased and flowering time is delayed. The delayed-flowering phenotype occurs not only under long-day but also under short-day conditions and can be overcome by vernalization, confirming that these components function in the autonomous pathway. Furthermore, we found that the complex components are involved in the regulation of multiple histone modifications at *FLC* chromatin. The identification and characterization of the AuPC in this study contribute to understanding of how the autonomous pathway regulates flowering time.

## MATERIALS AND METHODS

### Plant materials, growth conditions and flowering time analyses

All plant materials used in this study were in the Col-0 background. The T-DNA insertion mutants of *fld* (SalK_075401), *ld* (Salk_003039), *sdg26* (Salk_013895), *aprf1-9* (WiscDsLox489-492K11) and *flc* (SALK_003346) were obtained from the Arabidopsis Biological Resource Center. The mutants of *efl2* and *efl4* were generated by CRISPR/Cas9 using guide RNA 5′-GGAGATGTGTATTCTGGATT-3′ and 5′-GGAGATGTGTTGTCAGGATT-3′, respectively, in the pHEE401E-CRISPR/Cas9 system (45). The transgenic plants expressing FLD, LD, SDG26, EFL2, EFL4 and APRF1 were separately produced, by cloning the full-length genomic sequences of these genes under the control of their native promoters into the modified *pCAMBIA1305* vector and introducing the constructs into wild-type (WT) plants (Col-0) and mutant plants. 3×Flag or 3×HA was fused to the C-terminal of these proteins for western blot detection and affinity purification. Primers used for cloning these genes are listed in Supplementary Dataset S5. Unless otherwise specified, all plants used in this study were grown at 23°C under long-day conditions with a 16 h light/8 h dark photoperiod.

For flowering time analyses, numbers of rosette leaves produced by plants grown under long-day (16 h light/8 h dark) and short-day (8 h light/16 h dark) conditions were evaluated. For vernalization treatment, seeds were germinated on MS plates at 23°C for 3 days and then grown at 4°C for 50 days. After treatment, plants were transplanted to soil and grown at 23°C under long-day conditions.

### Affinity purification, mass spectrometry, co-IP and gel filtration

For affinity purification, 3–5 g of inflorescence of *3×Flag*-tagged *FLD*, *SDG26*, *LD*, *EFL2*, *EFL4* and *APRF1* transgenic plants in WT and mutant (*fld*, *sdg26* and *ld*) backgrounds were collected and ground into fine powder in liquid nitrogen. The powder was homogenized in 15–20 ml of lysis buffer [50 mM Tris–HCl (pH 7.6), 150 mM NaCl, 5 mM MgCl_2_, 10% glycerol, 0.1% NP-40, 0.5 mM DTT, 1 mM PMSF and one protease inhibitor cocktail tablet per 50 ml (Roche)] in 50-ml Falcon tubes by rotating at 4°C. After centrifugation, the supernatant was transferred to a new tube and was incubated with 100 μl of ANTI-FLAG M2 Affinity Agarose Gel (Sigma, A2220) at 4°C for 2.5 h. The agarose gel was washed five times with lysis buffer, and the proteins bound to the agarose gel were then eluted by incubation with 3×Flag peptides (Sigma, F4799) at 4°C for 30 min. Finally, the purified proteins were analyzed by mass spectrometry as described previously (46).

For co-immunoprecipitation (co-IP), after transgenic plants expressing Flag-tagged and HA-tagged proteins were crossed, the proteins from ∼1 g of 2-week-old seedlings of the F_1_ plants were extracted and immunoprecipitated by ANTI-FLAG M2 Affinity Agarose Gel (Sigma, A2220). The immunoprecipitated proteins were detected by western blotting using Flag antibody (Sigma, F7425) and HA antibody (Abcam, ab9110).

Gel filtration was performed as described previously (46). In brief, 0.5 g of seedlings of *FLD/SDG26/LD-3×Flag* transgenic plants in the WT background was ground and resuspended in 2 ml of lysis buffer, and the suspension was centrifuged at maximum speed at 4°C. The supernatant was passed through a 0.22‐μm filter, and 500 μl of the filtrate was loaded onto a Superose 6 (10/300GL) (GE Healthcare, 17-5172-01). Fractions were collected and detected by western blotting using Flag antibody (Sigma).

### Yeast two-hybrid

The full-length cDNA sequences of *FLD*, *LD*, *SDG26*, *SDG4*, *SDG7*, *SDG24*, *APRF1*, *ELF4*, *EFL1*, *EFL2*, *EFL3* and *EFL4* as well as the truncated forms of *FLD*, *LD* and *SDG26* were separately cloned in frame with the GAL4 activation domain (AD) in *pGADT7* and/or with the GAL4 DNA-binding domain (BD) in *pGBKT7*. First, the *pGADT7* and *pGBKT7* plasmids were linearized by EcoRI and BamHI digestion, and the inserts were amplified by PCR using chimeric primers (Supplementary Dataset S5) that contain a 20–25-bp homology to these cDNA sequences and a 15–16-bp homology to the linear ends of the plasmids. Second, the recombination between the linearized plasmids and the inserts was mediated by using the ClonExpress II One Step Cloning Kit (Vazyme Biotech, C112).

The *pGADT7* and *pGBKT7* constructs were transformed into the yeast strains AH109 and Y187, respectively. The transformed AH109 and Y187 were grown on synthetic dropout medium lacking Leu (SD-Leu) and lacking Trp (SD-Trp), respectively. Next, the positive colonies of the transformed AH109 and Y187 were mixed together in the YPD medium for 16–20 h, and the mated cells were then grown on dropout medium lacking Leu and Trp (SD-Leu/Trp). Subsequently, the positive colonies on SD-Leu/Trp were spotted on dropout medium lacking Trp, Leu and His (SD-Leu/Trp/His). Growth of mated cells on SD-Leu/Trp/His indicates an interaction between the combined AD fusion protein and BD fusion protein. 3-Amino-1,2,4-triazole was applied to reduce the background growth on SD-Leu/Trp/His.

### Protein expression and purification

The cDNA sequences encoding full-length EFL2, EFL4, FLD and SDG26 as well as the N-terminal (1–240 aa) and the C-terminal (320–492 aa) of SDG26 were cloned into *pGEX-6P-1* in frame with GST by using the ClonExpress II One Step Cloning Kit. These GST fusions were transformed and expressed in the *Escherichia coli* expression strain Transetta (DE3). GST-tagged proteins were purified from DE3 cultures with Glutathione Sepharose^®^ 4B (GE Healthcare, 17-0756-01). The full-length cDNA sequences of *LD*, *EFL2* and *EFL4* in frame with 3×Flag were cloned into *pAT424* between SalI and NotI restriction sites under the control of the *pTDH3* promoter by using the ClonExpress II One Step Cloning Kit. The *pAT424* constructs were introduced and expressed in the yeast strain YPH499 (47). Similarly, the full-length cDNA sequence of *APRF1* in frame with Myc tag was assembled into the *pAT424*-*LD-3*×*Flag* construct between FseI and AvrII restriction sites. Then, *LD-3*×*FLAG* and *ARPF1-Myc* were co-expressed by introducing the final construct containing both *LD-3*×*Flag* and *APRF1-Myc* in the yeast YPH499. The transformed YPH499 cells were cultured in SD-Trp medium and collected by centrifugation. After one wash with ice-cold water and one wash with Buffer A [50 mM Tris–HCl (pH 7.4), 1 mM EDTA, 150 mM NaCl and 10% glycerol], the cells were ground into powder in liquid nitrogen and lysed in fresh Buffer B [50 mM Tris–HCl (pH 7.4), 1 mM EDTA, 150 mM NaCl, 10% glycerol, 1 mM PMSF, 1 mM DTT, EDTA-free protease inhibitor cocktail (Roche) and 0.05% NP-40]. Subsequently, Flag-tagged LD, EFL2 and EFL4 were purified by using ANTI-FLAG M2 Affinity Agarose Gel (Sigma, A2220). The *pAT424* plasmid (NBRP ID: BYP7586) and the yeast strain YPH499 (NBRP ID: BY21467) were provided by Yeast Genetic Resource Center/National BioResource Project (NBRP), Japan. Primers used for cloning the constructs are listed in Supplementary Dataset S5.

### Pull-down assay

The purified Flag-tagged protein was mixed with the purified GST-tagged protein (or Myc-tagged APRF1) in 1 ml of pull-down buffer [20 mM Tris–HCl (pH 7.6), 150 mM NaCl, 1 mM DTT and EDTA-free protease inhibitor cocktail (Roche)]. A 100-μl volume of the mixture was used as input, and the remaining mixture was incubated with 40 μl of Glutathione Sepharose^®^ 4B at 4°C with gentle rotation for 1 h. After the Glutathione Sepharose^®^ 4B was washed six times with 1 ml of pull-down buffer at 4°C, proteins bound on the beads were eluted with 200 μl of elution buffer [50 mM Tris–HCl (pH 8.0), 150 mM NaCl, 20 mM glutathione and 1 mM DTT] at 4°C for 30 min. Finally, the eluted and input samples were boiled with SDS loading buffer and separated on SDS-PAGE gels for western blotting with Flag antibody (Sigma, F7425) and GST antibody (Abmart, M20007) or Myc antibody (Abmart, M20002).

### EMSA

The double-stranded DNA probe was generated by PCR using synthesized oligos. For electrophoretic mobility shift assay (EMSA), 1 μg of each purified protein was incubated with the DNA probe in the binding buffer [25 mM HEPES (pH 7.6), 50 mM KCl, 0.1 mM EDTA (pH 8.0), 12.5 mM MgCl_2_, 1 mM DTT, 0.5% (w/v) bovine serum albumin and 5% (w/v) glycerol] at 25°C for 30 min. Then, the binding reaction mixture was loaded onto a 4% nondenaturing polyacrylamide gel at 80 V for 3 h, and the bound DNA signals were visualized by ethidium bromide staining. The synthesized oligos are listed in Supplementary Dataset S5.

### Analysis of RNA transcripts

The transcript levels of *FLC*, *FT* and *SOC1* were measured by reverse transcription qPCR. First, total RNA was extracted with TRIzol reagent from 10-day-old seedlings grown under long-day conditions at 23°C. Second, the cDNAs were synthesized by using the 5× All‐In‐One RT Master Mix (with an AccuRT Genomic DNA Removal Kit) (Abm, G492). Finally, quantitative PCR was carried out on Bio-Rad CFX96 Real-Time System using KAPA SYBR^®^ FAST qPCR Kit Master Mix (2×) Universal (Kapa Biosystems, KR0389). A 0.1 μg quantity of cDNA was used in 20 μl reaction volume for 39 cycles in Hard-Shell PCR Plates (Bio-Rad, hsp9601). Three technical replicates were analyzed for each reaction. *ACT2* was also amplified as a reference gene, and primers for qRT-PCR are listed in Supplementary Dataset S5.

### RNA deep sequencing and data analysis

Total RNA was extracted with TRIzol reagent (Invitrogen) from 10-day-old seedlings of *fld*, *ld*, *sdg26 aprf1-9*, *efl2 efl4* and Col-0 grown under long-day conditions at 23°C. The libraries were generated and sequenced by BGISEQ-500 at Beijing Genomics Institute. For data analysis, the clean reads were aligned to the *Arabidopsis* genome (TAIR10) using HISAT2 (v2.1.0) (48). The reads mapped on the exon were counted using featureCounts (v1.6.4) (49). The differentially expressed genes (DEGs) were identified with |log_2_[fold change (FC) of reads per kilobase per million mapped reads (RPKM) between the mutant and the WT]| ≥ 1 and *P*-value <0.01 using edgeR (50). The R package venneuler was used to analyze the overlapped genes between different mutants for Venn diagrams. Heatmaps were drawn using log_2_FC by R package gplots.

### ChIP-PCR, ChIP-seq and data analysis

The enrichment of the histone modifications, including H3Ac, H3K4me2, H3K4me3, H3K36me3 and H3K27me3, on chromatin was detected by chromatin immunoprecipitation combined with sequencing (ChIP-seq), as described previously (51). For ChIP assays, a 2 g quantity of 10-day-old seedlings was harvested and ground into a fine powder in liquid nitrogen. The powder was suspended in 15 ml of nuclear extraction buffer [20 mM Tris–HCl (pH 7.5), 20 mM KCl, 2 mM EDTA (pH 8.0), 2.5 mM MgCl_2_, 25% glycerol, 250 mM sucrose, 5 mM DTT, 1% protease inhibitor cocktail tablet (Roche) and 1 mM PMSF] and was then subjected to cross-linking with 1% formaldehyde (Sigma, F8775). The sample was cross-linked for 20 min, and a concentration of 0.125 M glycine was added to stop the reaction. The mixture was then passed through two layers of Miracloth and centrifuged at 1500 × *g* for 20 min. The nuclei were washed in nuclear resuspension buffer [20 mM Tris–HCl (pH 7.5), 2.5 mM MgCl_2_, 25% glycerol and 0.2% Triton X-100] and then sonicated in sonication buffer [20 mM Tris–HCl (pH 8.0), 2 mM EDTA (pH 8.0), 0.2% NP-40, 1 mM PMSF and 1% protease inhibitor cocktail table] for 37 cycles (30 s on, 30 s off) by Bioruptor (Diagenode, Liege, Belgium). The sheared chromatin was diluted 1-fold with dilution buffer [2 mM EDTA (pH 8.0), 20 mM Tris–HCl (pH 8.0), 200 mM NaCl, 1 mM PSMF and 1% protease inhibitor cocktail] and incubated with H3Ac antibody (Millipore, 06-599), H3K4me3 antibody (Abcam, ab8580), H3K27me3 antibody (Millipore, 07-449) and H3K36me3 antibody (Abcam, ab9050) coupled with Dynabeads Protein A (Invitrogen, 100-01D) at 4°C overnight. The beads were washed five times with wash buffer [150 mM NaCl, 20 mM Tris–HCl (pH 8.0), 2 mM EDTA (pH 8.0), 0.1% Triton X-100 and 1 mM PMSF] and then with TE buffer [10 mM Tris–HCl (pH 8.0) and 1 mM EDTA (pH 8.0)]. The bead-bound DNA–protein complexes were eluted from the beads and reverse cross-linked with NaCl and Proteinase K (Sigma, P4850). The chromatin DNA was extracted using phenol/chloroform/isoamyl alcohol and was then subjected to library construction.

ChIP for ELF2-GFP, APRF1-GFP, SDG26-GFP and SDG26-truncated-GFP was carried out as previously described (52), with minor modifications. Briefly, 2 g of 10-day-old seedlings were harvested and soaked in Buffer I [0.4 M sucrose, 10 mM Tris–HCl (pH 8.0), 10 mM MgCl_2_, 0.1 mM PMSF, 1 mM DTT and 1% proteinase inhibitor cocktail tablets (Roche)] supplemented with 1% formaldehyde (Sigma). The materials were cross-linked for 12 min under vacuum conditions, and 0.125 M glycine was added to stop the reaction. Cross-linked samples were ground to a powder in liquid nitrogen, suspended in 20 ml of Buffer I and then subjected to rotation at 4°C for 20 min. The mixture was then passed through two layers of Miracloth and centrifuged at 1500 × *g* for 20 min. The nuclei pellet was washed with Buffer II [0.25 M sucrose, 10 mM Tris–HCl (pH 8.0), 10 mM MgCl_2_, 1% Triton X-100, 0.1 mM PMSF, 1 mM DTT and 1% proteinase inhibitor cocktail tablets]. The washed pellet was resuspended in 1.2 ml of Buffer III [1.7 M sucrose, 10 mM Tris–HCl (pH 8.0), 2 mM MgCl_2_, 0.15% Triton X-100, 0.1 mM PMSF, 1 mM DTT and 1% proteinase inhibitor cocktail tablets], and then centrifuged at 13 000 × *g* at 4°C for 1 h. The nuclei suspended in sonication buffer [50 mM Tris–HCl (pH 8.0), 10 mM EDTA (pH 8.0), 0.33% SDS and 1% proteinase inhibitor cocktail tablets] were sonicated for 21 cycles (30 s on, 30 s off) by Bioruptor (Diagenode, Liege, Belgium). Sonicated samples were centrifuged at 13 000 × *g* and diluted 2.3-fold with dilution buffer [16.7 mM Tris–HCl (pH 8.0), 1.2 mM EDTA (pH 8.0), 167 mM NaCl, 1.1% Triton X-100, 0.1 mM PMSF, 1 mM DTT and 1% proteinase inhibitor cocktail tablets], and then incubated with GFP-binding protein coupled with agarose beads (Cytvita, 17-096-01) overnight. Beads were sequentially washed with low-salt buffer [150 mM NaCl, 0.1% SDS, 1% Triton X-100, 2 mM EDTA (pH 8.0) and 20 mM Tris–HCl (pH 8.0)], high-salt buffer [500 mM NaCl, 0.1% SDS, 1% Triton X-100, 2 mM EDTA (pH 8.0) and 20 mM Tris–HCl (pH 8.0)], LiCl buffer [0.25 M LiCl, 1% NP-40, 1% sodium deoxycholate, 1 mM EDTA (pH 8.0) and 10 mM Tris–HCl (pH 8.0)] and TE buffer [1 mM EDTA (pH 8.0) and 10 mM Tris–HCl (pH 8.0)]. The beads were eluted with elution buffer (1% SDS and 0.1 M NaHCO_3_) at 65°C for 15 min. DNA was extracted from the eluted sample with the phenol/chloroform/isoamyl (25:24:1) reagent. Purified DNA was subjected to qPCR.

ChIP-seq libraries were constructed by Novogene (Tianjing, China) using the NEBNext^®^ Ultra™ DNA Library Prep Kit for Illumina^®^ (NEB, USA) and were sequenced by Illumina-NovaSeq (sequencing method: PE150). For data analysis, the clean reads were aligned to the *Arabidopsis* genome (TAIR10) using Bowtie2 (v2.2.6) (53). SICER (v2.0) was used to identify ChIP-enriched peaks between the WT and the *fld* mutant (54). Differentially expressed peaks in all mutants were identified based on the change of read counts (|log_2_FC| ≥ 0.585 and *P*-value <0.01) at peaks, and the read counts were normalized to RPKM by the number of clean reads mapped to the genome in each library. When the log_2_FC value is 0.585, the FC is 1.5. Therefore, |log_2_FC| ≥ 0.585 was used as the standard to identify differentially expressed peaks. The boxplots and bar plots were drawn with R package ggplot2. Heatmaps were drawn with R package gplots.

## RESULTS

### Identification of an FLD-containing protein complex

**Figure 1.**
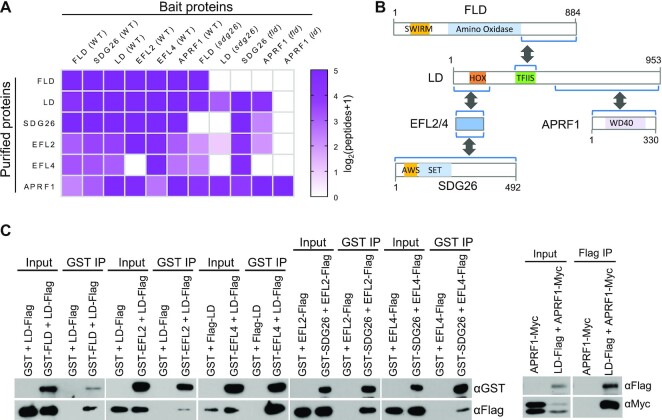
Identification and characterization of a multi-subunit complex containing the autonomous pathway components FLD, LD and SDG26. (**A**) Heatmap showing proteins co-purified with Flag-tagged FLD, LD, SDG26, EFL2, EFL4 and APRF1 as determined by affinity purification and mass spectrometry. Transgenic plants in the WT and indicated mutant backgrounds were subjected to the analyses. The number of detected peptides was used to estimate the abundance of purified proteins. The color scale represents log_2_(peptides + 1). (**B**) Schematic representation of the interaction of FLD, LD, SDG26, EFL2, EFL4 and APRF1 as determined by yeast two-hybrid (Y2H) analysis. The interaction domains of FLD and LD were determined by using a series of truncated versions of the proteins. Related Y2H data are shown in Supplementary Figures S3 and S5. (**C**) The interaction of FLD, LD, SDG26, EFL2, EFL4 and APRF1 as determined by *in vitro* pull-down assays. GST-, Flag- and Myc-tagged proteins were expressed from either *E. coli* or yeast. The proteins with different tags were mixed for the pull-down assay followed by western blotting.

FLD is an important component of the autonomous flowering pathway in *Arabidopsis* (11). To investigate how FLD is involved in the regulation of flowering time, we performed AP–MS using transgenic plants harboring a native promoter-driven *FLD* transgene tagged by the *Flag* epitope. Because FLD and the other autonomous pathway components are generally expressed more in the inflorescence than in the vegetative tissue as indicated by *Arabidopsis* eFP browser (55), we purified proteins from the inflorescence for affinity purification. The AP–MS assay not only identified two known FLD-interacting proteins, LD and SDG26 (35), but also identified three previously uncharacterized components, including EFL2 and EFL4, two closely related ELF4-like proteins (EFLs) (56) and APRF1/S2LA, a homolog of the yeast COMPASS complex subunit SWD2 (57,58) (Figure 1A; Supplementary Dataset S1). To assess whether these proteins interact with each other, we generated transgenic plants harboring native promoter-driven *LD*, *SDG26*, *EFL2*, *EFL4* and *APRF1* transgenes tagged by the *Flag* epitope. As determined by AP–MS, FLD, LD, SDG26, EFL2 and/or EFL4 (EFL2/4), and APRF1 were all co-precipitated with each other (Figure 1A; Supplementary Dataset S1). The results suggest that FLD, LD, SDG26, EFL2/4 and APRF1 form a multi-subunit complex.

To determine pairwise interactions of the complex components, we generated transgenic plants expressing each of the complex components tagged with Flag and HA epitopes and then conducted genetic crossing between the transgenic plants with different tags. By using their F_1_ progeny, we conducted co-IP and found that all tested pairs of the complex components interact with each other (Supplementary Figure S1). We performed gel filtration for FLD, LD and SDG26 to determine whether these proteins form a high-molecular-weight complex. The result indicated that all of the proteins can be eluted at ∼443-kDa fractions (Supplementary Figure S2), supporting the notion that FLD, LD, SDG26, EFL2/4 and APRF1 form a multi-subunit complex in *Arabidopsis*.

### Characterization of the protein–protein interaction in the complex

We conducted Y2H assays to determine how the complex components interact. As determined by Y2H, LD interacts with FLD, EFL2, EFL4 and APRF1, while EFL2 and EFL4 interact with SDG26 (Figure 1B; Supplementary Figure S3A and B). In addition to EFL2 and EFL4, *Arabidopsis* has two other ELF4-like proteins, EFL1 and EFL3. Our Y2H assays indicated that, unlike EFL2 and EFL4, ELF4, EFL1 and EFL3 do not interact with SDG26 (Supplementary Figure S3B). As determined by sequence analyses, while EFL2 and EFL4 are highly similar, they are moderately related to EFL3 and are distantly related to ELF4 and EFL1 (Supplementary Figure S4A and B), suggesting that the functions of EFL2 and EFL4 differ from those of ELF4, EFL1 and EFL3.

We then generated a series of truncated versions of LD and FLD to determine which regions of LD and FLD are responsible for the interactions, indicating that the C-terminal uncharacterized region of FLD is responsible for interacting with LD (Figure 1B; Supplementary Figure S5A–C). In LD, the N-terminal homeodomain, the TFIIS domain and the previously uncharacterized C-terminal region are required for LD interactions with FLD, EFL2/EFL4 and APRF1, respectively (Figure 1B; Supplementary Figure S5A and C–E), suggesting that LD functions as a scaffold protein to integrate the other components into the complex. Of note, the Y2H result indicated that FLD interacts with the truncated LD versions LD-2 and LD-4 but not with LD-3 or LD-8, although all of these truncated LD versions contain the TFIIS domain. The failure to detect the interaction between FLD and LD-3 or LD-8 is possibly caused by the failure of the expression of LD-3 and LD-8 or by other technical limitations of Y2H. As determined by the Y2H assay, APRF1 interacts with the C-terminal region of LD but not with the full-length LD (Supplementary Figure S5A and D). It is possible that the N-terminal region of LD suppresses the interaction between APRF1 and the C-terminal region of LD, and that the suppressive effect is abrogated in the AuPC. To validate the interaction as determined by Y2H, we expressed all of the complex components tagged by GST, Flag or Myc epitopes in *E. coli* or in yeast (Supplementary Figure S6), and subsequently performed pull-down assays. The results indicated that LD interacts with FLD, EFL2, EFL4 and APRF1, while SDG26 interacts with EFL2 and EFL4 (Figure 1C), completely confirming the interactions as determined by Y2H.

To validate the interaction of the complex components *in vivo*, we introduced *sdg26* into the *FLD-Flag* and *LD-Flag* transgenic lines and performed AP–MS to determine whether *sdg26* affects proteins co-precipitated with FLD-Flag and LD-Flag. Except for SDG26, all of the other complex components were co-purified with FLD-Flag and LD-Flag in the *sdg26* mutant as well as in the WT (Figure 1A; Supplementary Dataset S1). Similarly, we determined whether *fld* affects proteins co-precipitated with SDG26-Flag and APRF1-Flag, and found that all of the complex components other than FLD were co-precipitated in the *fld* mutant (Figure 1A; Supplementary Dataset S1). These results are consistent with the interactions as determined by the Y2H and pull-down assays. We introduced *ld* into *APRF1-Flag* transgenic lines to determine whether depletion of LD affects the interaction of APRF1 with the other complex components. As determined by AP–MS, all of the complex components were co-precipitated with APRF1 in the WT but not in the *ld* mutant (Figure 1A; Supplementary Dataset S1). These results further confirmed that LD functions as a scaffold protein and is responsible for the recruitment of the other components into the complex.

### The complex promotes flowering through the autonomous pathway

**Figure 2.**
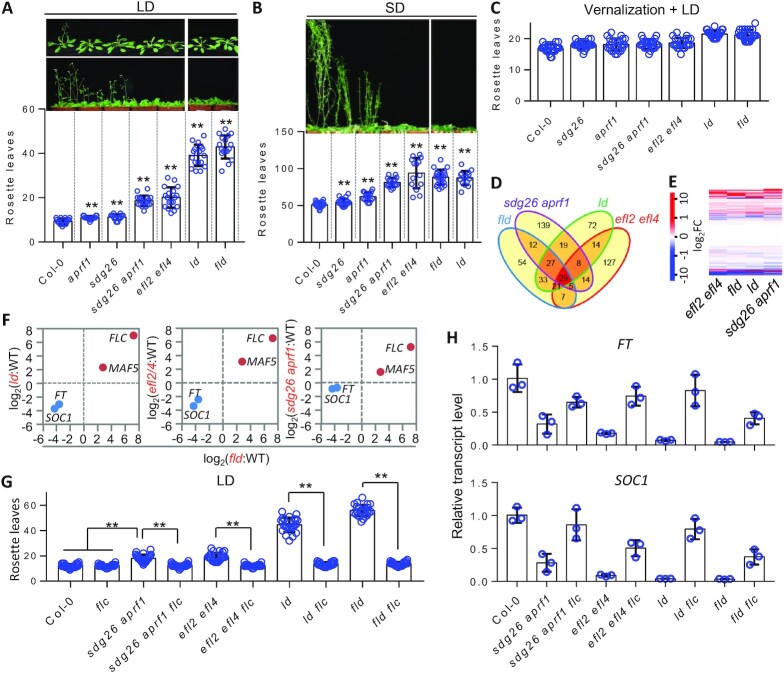
Components of the FLD-containing complex regulate flowering time and the expression of flowering time genes through the autonomous pathway. The flowering time of the mutants under long-day conditions (LD, 16 h light/8 h dark) (**A**) and short-day conditions (SD, 8 h light/16 h dark) (**B**). Bolting plants are shown at the top; the numbers of rosette leaves of the bolting plants are shown at the bottom. Under short-day conditions, the number of rosette leaves was counted even if the late-flowering mutant plants did not flower after 120 days of growth. Values are means ± standard deviation (*n* > 14). **P < 0.01, as determined by Student’s *t*-test. (**C**) The number of rosette leaves of vernalization-treated plants. Plants were grown under long-day conditions after the vernalization treatment (4°C for 50 days). Values are means ± standard deviation (*n* = 24). (**D–F**) Effects of indicated mutations on gene expression as determined by RNA-seq. DEGs in the mutants relative to the WT are indicated by Venn diagram (D), heatmap (E) and scatter plots (F). Genes were considered to be DEGs when log_2_(FC of RPKM between the mutant and the WT) > 1 or <−1 and *P* < 0.01. The color score shown in the heatmap and the value shown in the scatter plots are log_2_(FC of RPKM between the mutant and the WT). The effects of mutations on the expression of *FLC*, *MAF5*, *FT* and *SOC1* are shown by scatter plotting. (**G**) Effect of the *flc* mutation on the flowering time phenotype of the AuPC mutants under long-day conditions. The numbers of rosette leaves of bolting plants are shown as means ± standard deviation (*n* > 19). **P < 0.01, as determined by Student’s *t*-test. (**H**) Effect of *flc* mutation on the expression of *FT* and *SOC1* in the AuPC mutants. The expression of *FT* and *SOC1* was determined by quantitative PCR. Values are means ± standard deviation of three biological replicates.

The complex components FLD and LD are well-known components of the autonomous pathway (11,21); we therefore investigated whether all of the complex components function as a whole to promote flowering through the autonomous pathway. We created *efl2* and *efl4* single mutants by CRISPR/Cas9 and then generated the *efl2 efl4* double mutant by genetic crossing (Supplementary Figure S7A). The other mutants including *sdg26*, *aprf1*, *fld* and *ld* were available T-DNA mutants from the Arabidopsis Biological Resource Center (59); all the T-DNA mutants were validated by genotyping (Supplementary Figure S7B and C). Under long-day conditions (16 h light/8 h dark), the flowering time was markedly delayed in the *fld* and *ld* mutants and was weakly delayed in the *sdg26* and *aprf1* mutants (Figure 2A), which is consistent with previous reports that FLD, LD, SDG26 and APRF1 are responsible for the promotion of flowering (11,21,42,57). We also found that the flowering time was moderately delayed in the *efl2 efl4* mutant (Figure 2A). The late-flowering phenotype of the *efl2 efl4* mutant was partially complemented by either *EFL2-Flag* or *EFL4-Flag* transgenes, suggesting that EFL2 and EFL4 function redundantly in the regulation of flowering time (Supplementary Figure S8A and B). To confirm the function of SDG26 and APRF1 in the regulation of flowering time, we combined the *sdg26* and *aprf1* mutations by genetic crossing and found that the flowering phenotype of the *sdg26 aprf1* double mutant was further delayed compared to either *sdg26* or *aprf1* single mutant under long-day conditions (Figure 2A), suggesting that SDG26 and APRF1 function synergistically in the promotion of flowering.

As previously reported (6,60), the late-flowering phenotype of the autonomous pathway mutants is independent of photoperiod and can be suppressed by vernalization. We found that the late-flowering phenotype of all of the complex mutants was also evident under short-day conditions (8 h light/16 h dark) (Figure 2B), suggesting that the late-flowering phenotype of the complex mutants is independent of photoperiod. We also vernalized the mutants and WT plants and then grew them under long-day conditions, and found that the late-flowering phenotype of all of the mutants was completely suppressed by the vernalization treatment (Figure 2C). These results suggest that the complex components promote flowering time through the autonomous pathway.

Given that the autonomous pathway promotes flowering predominantly by suppressing the expression of the key flowering repressor gene *FLC* (6,11,61), we identified DEGs in the complex mutants by RNA deep sequencing (RNA-seq) and determined whether the late-flowering phenotype of the mutants was caused by an increase in *FLC* expression. Because *sdg26* and *aprf1* synergistically affect flowering time, the *sdg26 aprf1* double mutant was used in RNA-seq. From two independent replicates of the RNA-seq data, we identified 188, 223, 253 and 225 DEGs (log_2_FC > 1 or <−1; *P* < 0.01) in the *fld*, *ld*, *efl2 efl4* and *sdg26 aprf1* mutants, respectively (Figure 2D; Supplementary Dataset S2). The DEGs in these mutants were significantly overlapped (Figure 2D and E). In particular, we found that the expression of *FLC* and to a lesser extent the expression of the *FLC* homolog *MAF5* were increased in all of the complex mutants (Figure 2F; Supplementary Dataset S3), supporting the notion that the complex components promote flowering by reducing the expression of *FLC* and *MAF5*. Moreover, the RNA-seq analysis identified the critical flowering promotion genes *FT* and *SOC1* as co-downregulated genes in the *fld*, *ld* and *efl2 efl4* mutants (Figure 2F; Supplementary Figure S9; and Supplementary Dataset S3). Although *FT* and *SOC1* were not identified by RNA-seq as downregulated genes in the *sdg26 aprf1* mutant when stringent thresholds (log_2_FC < −1; *P* < 0.01) were used, the downregulation of *FT* (log_2_FC = −0.73; *P* = 0.025) and *SOC1* (log_2_FC = −0.90; *P* = 0.00019) was evident when the thresholds were slightly reduced (log_2_FC > 0.5 or <−0.5; *P* < 0.05) (Figure 2F; Supplementary Dataset S3). Therefore, the complex components not only suppress the expression of the flowering repressor genes *FLC* and *MAF5* but also promote the expression of the flowering promotion genes *FT* and *SOC1*.

Because *FLC* represses flowering by preventing the transcription of *FT* and *SOC1* (62–64), we determined whether the increased expression of *FLC* in the complex mutants is critical for the late-flowering phenotype and for the reduced expression of *FT* and *SOC1*. We introduced the *flc* mutation into the complex mutants by genetic crossing and found that the late-flowering phenotype of all of the mutants was eliminated by the *flc* mutation (Figure 2G). Consistent with the elimination of the late-flowering phenotype, the reduced expression of *FT* and *SOC1* in the *sdg26 aprf1*, *efl2 efl4*, *fld* and *ld* mutants was significantly rescued by the *flc* mutation (Figure 2H). These results suggest that the complex components suppress *FLC* expression and thereby promote flowering through the autonomous pathway. We hereafter refer to this collection of components as the AuPC.

### Identification of a DNA-binding coiled-coil domain in SDG26 that is responsible for the association of SDG26 with *FLC* chromatin

**Figure 3.**
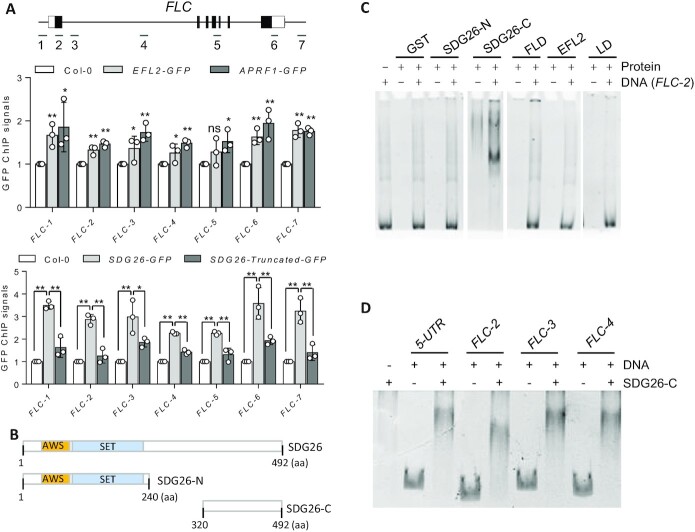
EFL2, APRF1 and SDG26 associate with *FLC* chromatin, and the C-terminal coiled-coil domain of SDG26 directly binds to double-stranded DNA. (**A**) Association of EFL2, APRF1 and SDG26 with *FLC* chromatin as determined by ChIP-PCR. The ChIP-PCR signals were detected at the indicated positions of *FLC*. Anti-GFP antibody was used for ChIP-PCR in WT and in *EFL2-GFP*, *APRF1-GFP* and *SDG26-GFP* transgenic plants. The transgenic plants expressing the C-terminal (320–492 aa) truncated SDG26-GFP were used to determine the effect of the C-terminal deletion on the association of SDG26 with *FLC* chromatin. Values are means ± standard deviation of three biological replicates. **P < 0.01, *P < 0.05; ns, not significant, as determined by Student’s *t*-test. (**B**) Schematic representation of the full-length SDG26 and the truncated versions SDG26-N (1–240 aa) and SDG26-C (320–492 aa). (**C**) The binding of SDG26, FLD, EFL2 and LD to DNA as determined by EMSA. SDG26-N (1–240 aa), SDG26-C (320–492 aa), FLD and EFL2 were fused with GST and were expressed and purified from *E. coli*. LD fused with Flag was expressed and purified from yeast. The sequence of the DNA probe is from the SDG26-bound *FLC* chromatin locus. (**D**) The binding of SDG26 to different DNA probes as determined by EMSA. SDG26-C fused with GST was expressed and purified from *E. coli*. The DNA probes are from different *FLC* loci as indicated.

**Figure 4.**
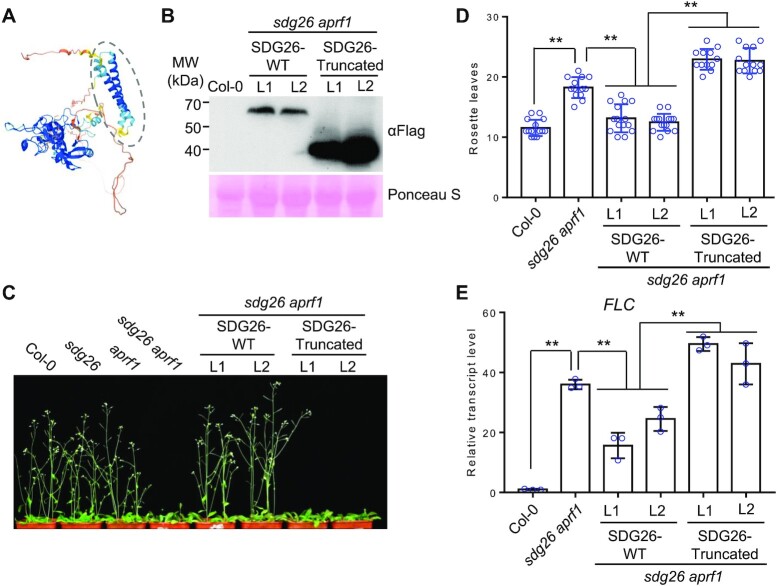
Effect of the C-terminal deletion on the regulation of flowering time and *FLC* expression by SDG26. (**A**) Structure of SDG26 as predicted by AlphaFold. The coiled-coil domain composed of two alpha helices is labeled by a dashed oval. (**B**) Expression levels of full-length and C-terminal truncated SDG26 proteins in corresponding transgenic plants as determined by western blotting. Two individual transgenic lines were subjected to the analysis. The ribosome protein stained by Ponceau S is indicated as a loading control. (**C**, **D**) Restoration of the late-flowering phenotype of the *sdg26 aprf1* mutant by full-length and C-terminal truncated versions of SDG26. The morphological phenotype (C) and the number of rosette leaves (D) in indicated genotypes are shown. Values are means ± standard deviation (*n* > 12). **P < 0.01, as determined by Student’s *t*-test. (**E**) Restoration of the *FLC* expression of the *sdg26 aprf1* mutant by the full-length and truncated *SDG26* transgenes. Values are from three independent biological replicates. **P < 0.01, as determined by Student’s *t*-test.

Among the AuPC components, FLD was previously reported to associate with *FLC* chromatin as determined by chromatin immunoprecipitation combined with PCR (ChIP-PCR) (32). We generated *EFL2-GFP*, *APRF1-GFP* and *SDG26-GFP* transgenic plants and determined the association of EFL2, APRF1 and SDG26 with *FLC* chromatin by ChIP-PCR; the results indicated that these components are enriched at the full length of *FLC* and especially at the 5′- and 3′-ends of the gene body (Figure 3A), suggesting that the AuPC components function as a whole to associate with the *FLC* chromatin. We then determined which components of the AuPC can directly bind to DNA. SDG26, EFL2, FLD and LD were expressed and purified from *E. coli* or yeast and were then subjected to an EMSA. The results indicated that the C-terminal uncharacterized region (320–492 aa) of SDG26 bound to the double-stranded DNA from the *FLC* sequence (*FLC-2*), whereas EFL2, FLD and LD did not bind to the DNA sequence (Figure 3B and C). According to the protein structure of SDG26 predicted by AlphaFold (65), the C-terminal of SDG26 forms a coiled-coil domain composed of two alpha helices (Figure 4A), a major domain that is responsible for binding to DNA (66), suggesting that the coiled-coil domain is responsible for the binding of SDG26 to DNA. We further found that the C-terminal of SDG26 bound to DNA from different *FLC* loci (Figure 3D), indicating that the C-terminal of SDG26 binds to DNA in a sequence-independent manner. It is possible that SDG26 enhances the basic binding ability of AuPC for *FLC* but is not required for the binding specificity of AuPC for different *FLC* loci.

We therefore expressed a C-terminal truncated version of SDG26 in *Arabidopsis* plants and assessed the effect of the truncation on the association of SDG26 with *FLC* chromatin by ChIP-PCR. We found that the truncation significantly reduced the association of SDG26 with *FLC* chromatin (Figure 3A). We also introduced the C-terminal truncated SDG26 into the *sdg26 aprf1* mutant for complementation testing (Figure 4B). Given that the effect of *sdg26* on flowering time is more significant in the *aprf1* mutant background than in the WT background (Figure 2A and B), we performed complementation testing for WT and truncated *SDG26* in the *sdg26 aprf1* double mutant rather than in the *sdg26* single mutant. We found that the late-flowering phenotype of the *sdg26 aprf1* mutant was significantly complemented by the WT SDG26 but not by the C-terminal truncated SDG26 (Figure 4C and D). Consistent with the effect of the truncation on the function of SDG26 in flowering time regulation, the *FLC* expression level was also significantly restored by the WT SDG26 but not by the C-terminal truncated SDG26 (Figure 4E). The C-terminal coiled-coil domain of SDG26 is conserved in the SDG26 orthologs of other angiosperms but not in any other TrxG histone methyltransferases (Supplementary Figure S10), suggesting that the DNA-binding coiled-coil domain represents a unique property of SDG26 and its orthologs in angiosperms. The truncated *SDG26* transgene seems to weakly enhance the late-flowering phenotype and *FLC* expression in the *sdg26 aprf1* mutant background (Figure 4D and E), indicating that the truncated SDG26 has a dominant negative effect on the AuPC. Together, these results indicate that the binding of the SDG26 coiled-coil domain to DNA mediates the association of SDG26 with *FLC* chromatin and thereby promotes flowering by suppressing *FLC* expression.

### The regulation of flowering time by the AuPC is independent of the histone methyltransferase activity of SDG26

**Figure 5.**
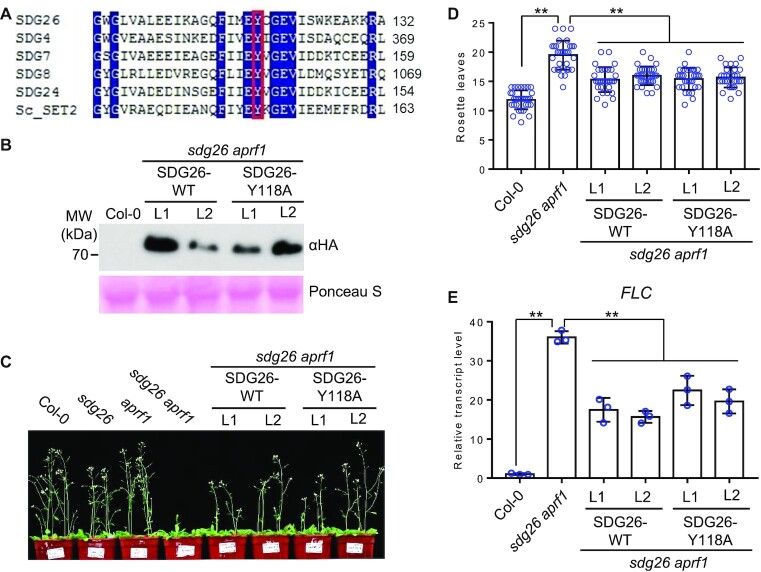
Effect of the SET domain mutation on the regulation of flowering time and *FLC* expression by SDG26. (**A**) Alignment of the conserved SET domain sequences in SDG26 and its paralogs in *Arabidopsis* and in the closely related SET2 in yeast. The tyrosine site responsible for the histone methyltransferase activity is highlighted by a box. (**B**) The expression levels of WT SDG26 and mutated SDG26 (Y118A) in corresponding transgenic plants as determined by western blotting. Two individual transgenic lines were subjected to the analysis. The ribosome protein stained by Ponceau S is indicated as a loading control. (**C**, **D**) Restoration of the late-flowering phenotype of the *sdg26 aprf1* mutant by the WT and mutated SDG26 transgenes. The morphological phenotype (C) and the number of rosette leaves (D) in indicated genotypes are shown. Values are means ± standard deviation (*n* > 30). **P < 0.01, as determined by Student’s *t*-test. (**E**) Restoration of the *FLC* expression of the *sdg26 aprf1* mutant by the WT and mutated *SDG26* transgenes. Values are from three independent biological replicates. **P < 0.01, as determined by Student’s *t*-test.

SDG26 was previously reported to activate the expression of the flowering promotion gene *SOC1* by facilitating H3K4me3 and H3K36me3 (43,44). Given that SDG26 is a member of the TrxG histone methyltransferase family and has active histone methyltransferase activity as determined by an *in vitro* assay (42), the histone methyltransferase activity of SDG26 is likely to be essential for mediating H3K4me3 and H3K36me3 at *SOC1* and thereby promotes flowering. In an independent study (35), however, the histone methyltransferase activity of SDG26 was not detected by an *in vitro* assay. These inconsistent results prompted us to determine whether the histone methyltransferase activity of SDG26 is involved in the regulation of flowering time. SDG26-Y118 is conserved in the SET domains of histone methyltransferases and has been shown to be critical for the catalytic activity of the SDG26 homolog in yeast as determined by a structural analysis (67). Thus, we generated a SDG26-Y118 to A (SDG26-Y118A) mutation that disrupted the critical residue in the conserved catalytic domain of SDG26 (Figure 5A), and then introduced the mutated *SDG26* into the *sdg26 aprf1* mutant plants for complementation testing. Although the expression levels of the mutated *SDG26* were similar to those of the WT *SDG26* (Figure 5B), the late-flowering phenotype of the *sdg26 aprf1* mutant was restored by the mutated SDG26 as well as by the WT SDG26 (Figure 5C and D), indicating that the histone methyltransferase activity of SDG26 is not required for the SDG26-dependent promotion of flowering. Consistent with the promotion of flowering by the mutated *SDG26*, the *FLC* expression level was suppressed by the mutated *SDG26* as well as by the WT *SDG26* (Figure 5E). These results suggest that SDG26 functions as a subunit of the AuPC and thereby regulates flowering time in a histone methyltransferase activity-independent manner.

### The AuPC regulates multiple histone modifications at *FLC* and *MAF5* chromatin loci

**Figure 6.**
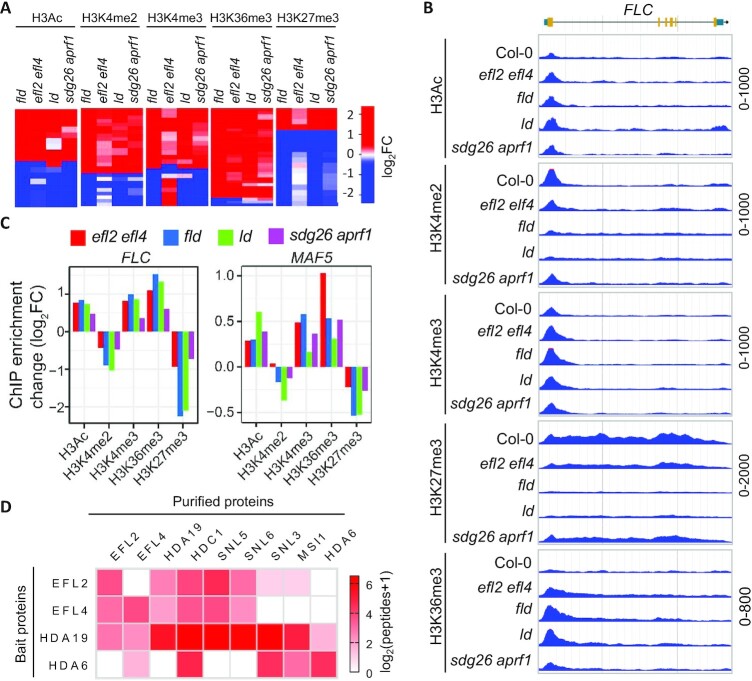
Effect of the AuPC mutations on H3Ac, H3K4me2, H3K4me3, H3K36me3 and H3K27me3 at the whole-genome level. (**A**) Heatmaps showing the effect of the AuPC mutations on histone modifications at genes with significantly up- and downregulated histone modifications in the *fld* mutant. The color scale denotes log_2_FC of ChIP-seq signals between mutants and WT. (**B**) Genome browser view of histone modifications at *FLC* in the AuPC mutants and the Col-0 control. ChIP-seq signals are shown for H3Ac, H3K4me2, H3K4me3, H3K27me3 and H3K36me3. The scale of RPKM is indicated for each panel. (**C**) Effect of the AuPC mutations on the levels of H3Ac, H3K4me2, H3K4me3, H3K36me3 and H3K27me3 at *FLC* and *MAF5*. The effect is indicated by log_2_FC of ChIP-seq signals between mutants and WT. The H3K27me3 level was determined by ChIP-seq signals in the full-length gene body, and the H3Ac, H3K4me2, H3K4me3 and H3K36me3 levels were determined by ChIP-seq signals at the transcription start site (−100 to +400 bp). (**D**) Heatmap showing proteins co-purified with Flag-tagged EFL2, EFL4, HDA19 and HDA6 as determined by affinity purification and mass spectrometry. The number of detected peptides was used to estimate the abundance of purified proteins. The color scale represents log_2_(peptides + 1).

As previously reported, the known autonomous pathway component FLD affects multiple histone modifications at *FLC* and *FLC* homologs (11,24,30–34). We therefore performed ChIP-seq analyses to determine whether all of the AuPC components identified in this study regulate flowering time by affecting these histone modifications. Based on two independent replicates of the ChIP-seq data, we identified genes with increased and reduced enrichments of the histone modifications in the AuPC mutants relative to the WT (Figure 6A; Supplementary Dataset S4). Interestingly, only a small number of genes (<100 for most of the histone modifications) showed significant changes (FC > 1.5, *P* < 0.01) in the enrichment of histone modifications in the AuPC mutants. As expected, the changes in histone modifications were highly similar among the different AuPC mutants (Figure 6A; Supplementary Dataset S4), supporting the inference that these AuPC components function as a whole to regulate histone modifications at the same target genes. The enrichments of all of the tested histone modifications were markedly altered at *FLC* chromatin in all of the AuPC mutants (Figure 6B and C; Supplementary Dataset S4). Consistent with the increased expression of *FLC* in the AuPC mutants, the histone modifications (H3Ac, H3K4me3 and H3K36me3) associated with transcriptional activation were increased, and the histone modification (H3K27me3) associated with transcriptional repression was reduced in the AuPC mutants (Figure 6B and C), suggesting that all of the AuPC components can suppress *FLC* expression at least partially by regulating these histone modifications.

The autonomous pathway component FLD was reported to function as an LSD1-like histone demethylase that is responsible for demethylating H3K4me1 and H3K4me2 but not H3K4me3 (24,68). Previous studies showed that the H3K4me2 level at the intragenic region of *FLC* was increased in the *fld* mutant, and the increased level of H3K4me2 was thought to be associated with the activation of *FLC* expression (24,32,33). Unlike these studies, recent studies indicated that H3K4me2 was enriched around the transcription start site of *FLC* in the WT and the enrichment was reduced in the *fld* mutant (35,36). Our H3K4me2 ChIP-seq results confirmed the enrichment of H3K4me2 at the *FLC* transcription start site and the reduction of H3K4me2 in the *fld* mutant (Figure 6B and C; Supplementary Table S1; and Supplementary Dataset S4). Moreover, the reduced enrichment of H3K4me2 was also found in the other AuPC mutants (Figure 6B and C; Supplementary Table S1; and Supplementary Dataset S4). A whole-genome analysis in rice indicated that H3K4me2 functions as a mark associated with transcriptional repression (69). We therefore predicted that the increased expression of *FLC* in the AuPC mutants is partially caused by the reduced level of H3K4me2, although how the H3K4me2 level is reduced in the AuPC mutants remains elusive. Considering that H3K4me3 is over-accumulated at the transcription start site of *FLC* in the AuPC mutants, we predict that the accumulation of H3K4me2 caused by depletion of FLD is rapidly converted into H3K4me3, resulting in a significant reduction in H3K4me2.

Among all of the tested AuPC mutants, we found that *MAF5* chromatin showed an increase in H3Ac, H3K4me3 and H3K36me3, and a reduction in H3K27me3 (Figure 6C; Supplementary Dataset S4); these changes are consistent with the increased expression of *MAF5* in the AuPC mutants (Figure 2F; Supplementary Dataset S2). The AuPC may therefore either directly or indirectly regulate these histone modifications at both *FLC* and *MAF5* and thereby suppresses the expression of these genes. The H3K4me2 level of *MAF5* chromatin was reduced in the AuPC mutants *fld*, *ld* and *sdg26 aprf1* and was not significantly affected in the *efl2 efl4* mutant (Figure 6C; Supplementary Dataset S4), suggesting that the increased expression of *MAF5* in the AuPC mutants cannot be explained by the H3K4me2 change. As shown by previous whole-genome analyses, although H3K4me3 is associated with the activation of gene expression, H3K4me2 is not an indicator of gene expression (70,71). We therefore infer that the AuPC suppresses gene expression at least partially by affecting the active histone modifications H3Ac, H3K4me3 and H3K36me3 and the repressive histone modification H3K27me3. In contrast, H3K4me2 is unlikely to be a casual effect of the AuPC on the expression of *FLC* and *MAF5*.

Two previous studies (35,36) indicated that the histone demethylase FLD mediates demethylation of H3K4me1 and thereby represses *FLC* expression. Given that the FLD is a core component of the AuPC, we infer that FLD-dependent demethylation of H3K4me1 is important for the regulation of *FLC* expression by the AuPC. It is possible that the AuPC primarily mediates demethylation of H3K4me1 and subsequently affects multiple other histone modifications. FLD was shown to mediate demethylation of H3K4me1 especially at convergent genes and thereby repress transcription (36). By re-analyzing the H3K4me1 data, we found that while FLD-dependent H3K4me1 demethylation is enriched at convergent genes, it also occurs at a number of tandem genes (Supplementary Figure S11A). Moreover, the upregulated DEGs in all the tested AuPC mutants were either not enriched or only slightly enriched at convergent genes (Supplementary Figure S11A). By comparing upregulated DEGs in the AuPC mutant and genes with increased levels of H3K4me1 in the *fld* mutant, we found that only a small portion of upregulated DEGs in the AuPC mutants showed increased levels of H3K4me1 in the *fld* mutant (Supplementary Figure S11B). These analyses implicate that the AuPC represses transcription not only via FLD-dependent H3K4me1 demethylation but also via other mechanisms.

To determine whether the AuPC can regulate histone modifications in an H3K4me1 demethylation-independent manner, we analyzed the proteins co-purified with the AuPC components based on AP–MS assays in *Arabidopsis* plants, and found that the histone deacetylase complex components HDA19, HDC1, SNL5 and SNL6 were co-purified with EFL2-Flag and EFL4-Flag (Figure 6D; Supplementary Dataset S1). Moreover, the AP–MS results in our recent study (72) indicated that EFL2 and/or EFL4 were co-purified with HDA6-Flag and HDA19-Flag. These results suggested that EFL2 and EFL4 interact with the histone deacetylase complex, supporting the interference that the AuPC represses transcription at least partially by mediating histone deacetylation. Moreover, previous studies showed that loss of the HDAC components not only causes an increased level of histone acetylation but also causes increased levels of H3K4me3 and H3K36me3 and a reduced level of H3K27me3 at the *FLC* chromatin (22,34,73–75), suggesting that the HDAC-dependent histone deacetylation can indirectly affect multiple other histone modifications. We therefore predict that the AuPC primarily mediates histone deacetylation and subsequently leads to alteration of other histone modifications. These results together provide insights into the molecular mechanism underlying the regulation of flowering time by the autonomous pathway.

## DISCUSSION

In the current study, we identified two ELF4-like proteins, EFL2 and EFL4, as components of the AuPC in *Arabidopsis*. ELF4 is an important component of the circadian clock that regulates various biological processes, including hypocotyl elongation, light-induced seedling de-etiolation and flowering time (76–78). ELF4 is required for the prevention of early flowering under noninductive photoperiod conditions (76). *Arabidopsis* has four ELF4-like proteins: EFL1, EFL2, EFL3 and EFL4 (56). By evaluating the flowering time of *EFL1*, *EFL2* and *EFL3* overexpression lines in the *elf4* mutant background, a previous study indicated that like *ELF4*, *EFL1* and *EFL3* are responsible for the suppression of flowering, whereas *EFL2* has no significant effect on flowering (56). Here, we found that, unlike ELF4, EFL1 and EFL3, which suppress flowering, EFL2 and EFL4 function redundantly as components of the newly identified AuPC and are responsible for the promotion of flowering through the autonomous pathway. These results suggest that, compared with ELF4, EFL1 and EFL3, their homologs EFL2 and EFL4 have an opposite effect on flowering. Although ELF4, EFL1 and EFL3 function as components of the circadian clock and prevent early flowering under noninductive photoperiod conditions, EFL2 and EFL4 function as components of the autonomous pathway to promote flowering. We predict that the antagonistic effects of these homologs coordinate the autonomous and photoperiod-dependent flowering pathways to ensure proper time of flowering under variable environmental conditions.

We demonstrated that several chromatin-associated autonomous pathway components form a multi-subunit complex that regulates histone modifications and transcription of *FLC*. However, it is largely unknown how the AuPC associates with specific chromatin loci, including *FLC*. SDG26 contains a previously uncharacterized C-terminal region, which is unique among TrxG histone methyltransferases in *Arabidopsis* but is conserved in the SDG26 orthologs of other plants. Our results suggest that the C-terminal coiled-coil domain of SDG26 binds to DNA *in vitro* and is responsible for the association of SDG26 with *FLC* chromatin *in vivo*. Given that SDG26 is a component of the AuPC, it is possible that the DNA-binding ability of SDG26 is partially responsible for the association of the AuPC with chromatin. However, because the effect of *sdg26* on flowering time is markedly weaker than that of the *fld*, *ld* and *efl2 efl4* mutations, SDG26 is unlikely to play a major role in mediating the association of the AuPC with chromatin. We predict that other components in the AuPC are also required for the association of the AuPC with chromatin. LD contains a putative homeodomain (79), which is potentially responsible for DNA binding. However, our DNA-binding assay failed to detect the binding of LD to DNA. Considering that the late-flowering phenotype is markedly enhanced in the *sdg26 aprf1* double mutant relative to either *sdg26* or *aprf1* single mutant, we speculate that APRF1 cooperates with SDG26 to mediate the association of the AuPC with chromatin. Future research should determine whether and how APRF1 cooperates with SDG26 to reinforce the association of the AuPC with *FLC* chromatin.

We found that the AuPC regulates multiple histone modifications, including H3Ac, H3K4me3, H3K36me3 and H3K27me3 at *FLC*, which is consistent with previous reports that the well-known autonomous pathway component FLD is required for maintenance of proper levels of multiple histone modifications at *FLC* (11,24,30–34). However, except for FLD, which mediates histone H3K4me1/2 demethylation, the other AuPC components do not directly regulate histone modifications at *FLC*. The mechanism by which the AuPC regulates multiple histone modifications remains to be determined. The histone marks H3K4me3 and H3K36me3 at *FLC* chromatin are directly mediated by the TrxG histone methyltransferases ATX1 or SDG25 and SDG8, respectively (39–41). CLF, a catalytic subunit of the PRC2 histone methyltransferase complex, is responsible for deposition of H3K27me3 at *FLC* chromatin in the rapid-flowering accessions (29). Interestingly, in the *atx1* and *sdg25* mutants, the reduction of H3K4me3 at *FLC* chromatin is accompanied by a reduced level of H3K36me3 and an increased level of H3K27me3; in the *clf* mutant, the reduction of H3K27me3 at *FLC* chromatin is accompanied by increased levels of H3K4me3 and H3K36me3 (29,39,80). FLD-dependent demethylation of H3K4me1 was recently shown to be required for repression of *FLC* transcription (35,36). These results suggest that changes in multiple histone modifications are coordinated to repress *FLC* transcription, although the underlying mechanism is largely unknown.

As determined by our current and previously published AP–MS data (72), we found that several components of the histone deacetylase complex (HDA6, HDA19, HDC1, SNL5 and SNL6) interact with the AuPC components EFL2 and/or EFL4, supporting the notion that the AuPC represses *FLC* transcription by mediating histone deacetylation. The histone deacetylase HDA6 not only regulates histone deacetylation but also affects multiple other histone modifications by directly associating with *FLC* chromatin (34,73,75). The histone deacetylase complex components HDA6 and FVE were reported to interact with FLD and to thereby mediate the coupling of histone deacetylation and H3K4me1/2 demethylation. Moreover, the autonomous pathway component FVE was shown to interact with the PRC2 histone H3K27 trimethyltransferase complex and to thereby mediate histone H3K27 trimethylation at *FLC* (22,74). The interactions between the autonomous pathway components and the different histone modifiers provide a plausible explanation for the effects of the AuPC on multiple histone modifications. Taken together, the results of the current study provide a framework for further investigations of the autonomous flowering pathway.

## DATA AVAILABILITY

Raw data of RNA-seq and ChIP-seq results have been deposited in Gene Expression Omnibus (accession number GSE193253).

## Supplementary Material

gkac551_Supplemental_Files
