# Supplementary material for: Characterization of an autonomous pathway complex that promotes flowering in Arabidopsis
Source: Nucleic Acids Res. 2022 Jun 29;50(13):7380–95. doi: 10.1093/nar/gkac551 (PMC9303297; doi:10.1093/nar/gkac551)
Supplement: gkac551_Supplemental_Files [file gkac551_supplemental_files.zip › Supplemental Figures.pdf]

# Supplemental Figure 1

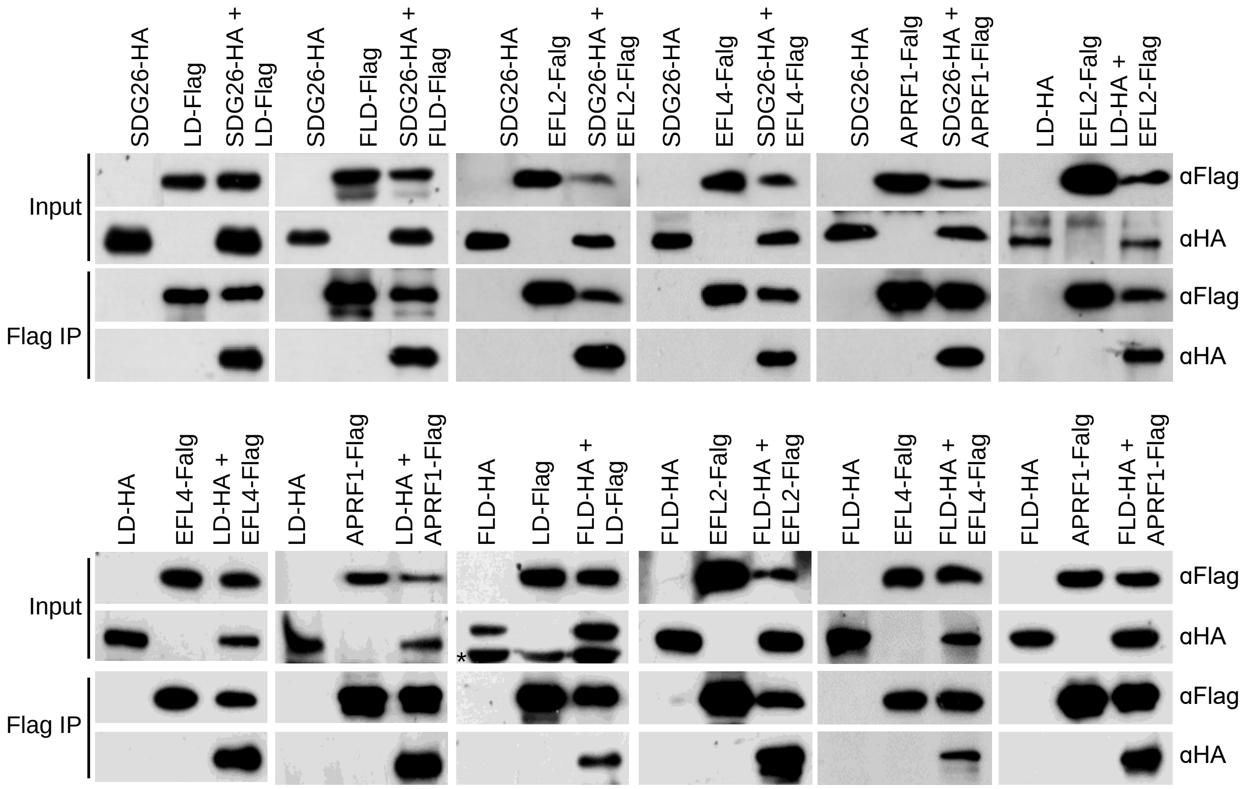


**Supplemental Figure 1. Pairwise interactions of FLD, LD, SDG26, EFL2, EFL4, and APRF1 as determined by co-IP.** Indicated Flag-tagged transgenic plants were crossed to HA-tagged transgenic plants. The progeny expressing both Flag- and HA-tagged proteins were used for co-IP.

# Supplemental Figure 2

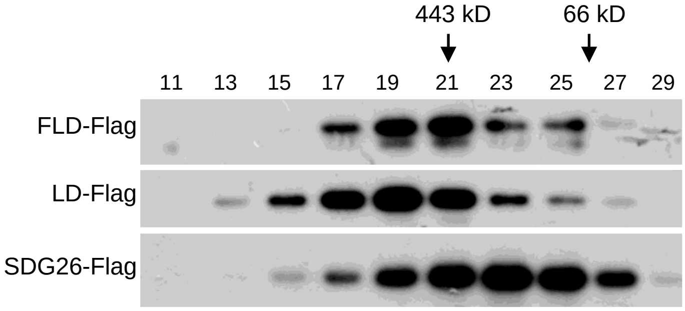


**Supplemental Figure 2. Determination of the high-molecular-weight complex containing FLD, LD, and SDG26 by gel filtration.** Total proteins extracted from *FLD-Flag*, *LD-Flag*, and *SDG26-Flag* transgenic plants were run on a Superose 6 (10/300 GL) column followed by western blotting. The 443-kD (ApoF, apoferritin) and 66-kD (Alb, albumin) mark proteins were used for estimation of protein size.

# Supplemental Figure 3

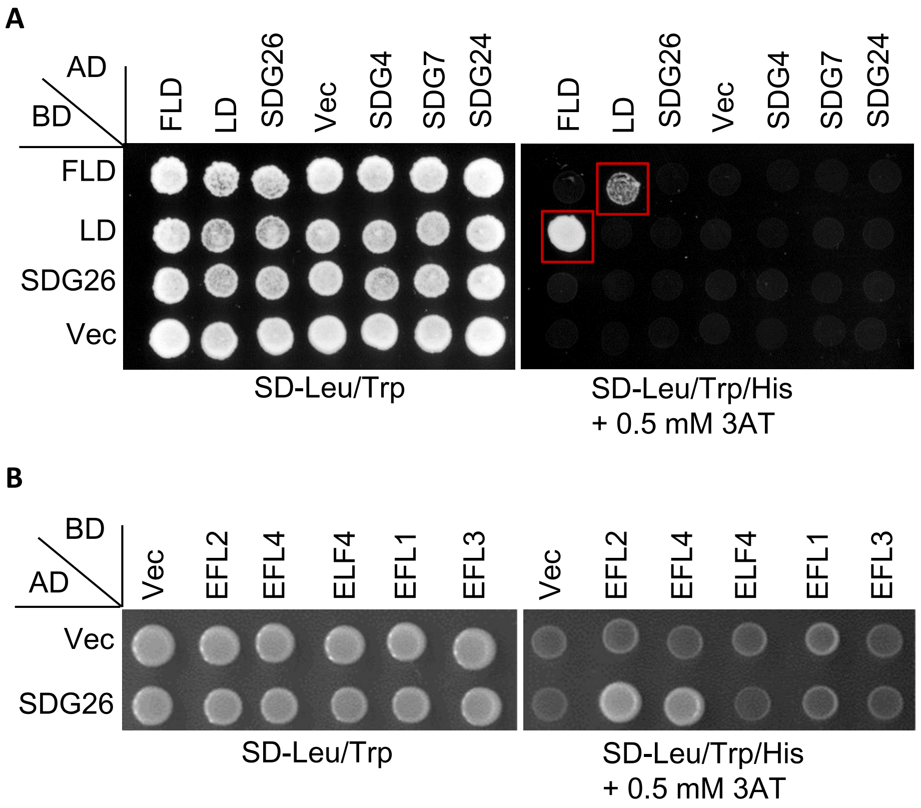


**Supplemental Figure 3. Determination of the interaction between components of the newly identified complex.** (A,B) Determination of the interaction of FLD, LD, SDG26, SDG4, SDG7, and SDG24 (A), and the interaction between SDG26 and ELF4, EFL1, EFL2, EFL3, or EFL4 (B) as determined by Y2H assays. The yeast strains harboring indicated pairs of GAL4-AD and GAL4-BD fused proteins were grown on SD medium minus Leu, Trp, and His (SD-Leu/Trp/His) supplemented with 3-AT for a growth assay. The same yeast strains were also grown on the SD medium minus Leu and Trp (SD-Leu/Trp) as a control.

# Supplemental Figure 4

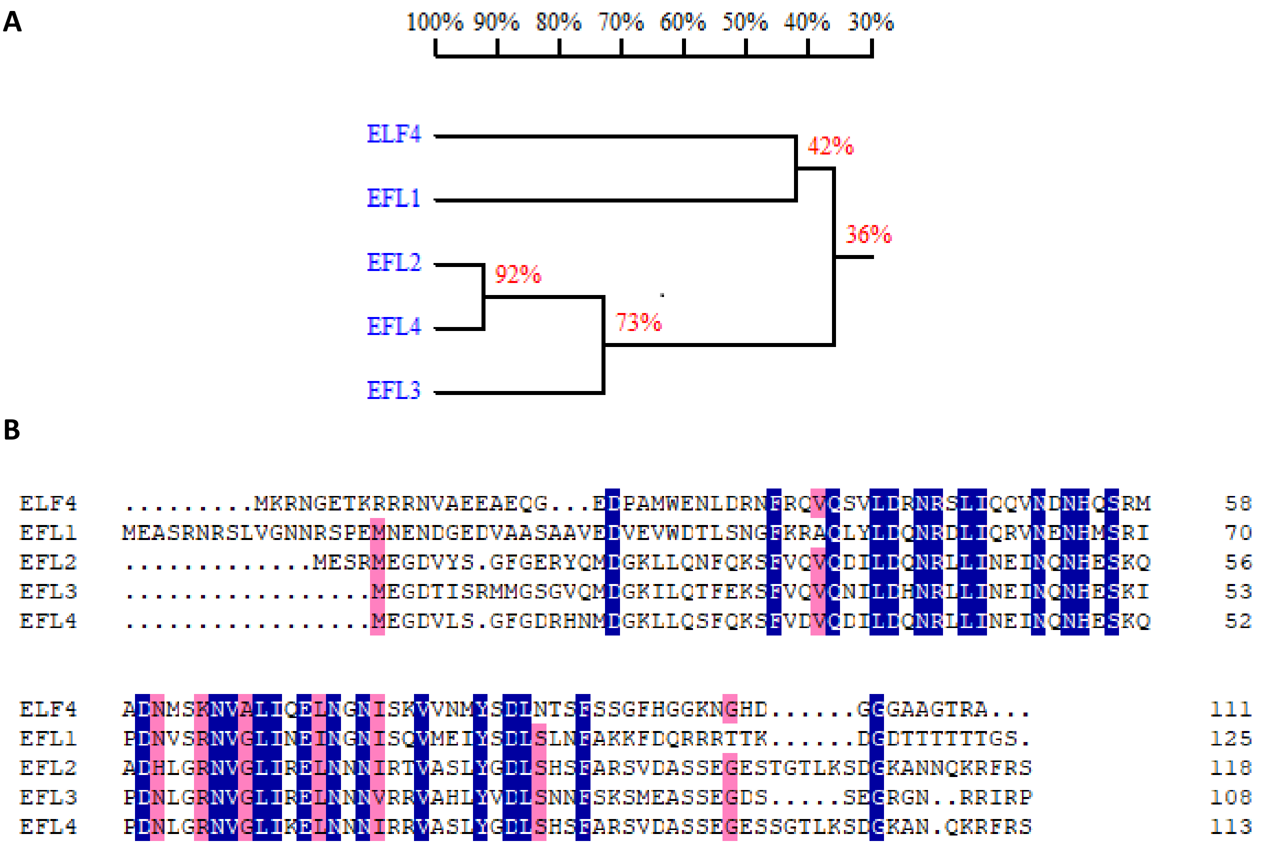


**Supplemental Figure 4. Sequence analyses of ELF4 and the ELF4-like proteins EFL1, EFL2, EFL3, and EFL4.** (A) Phylogenic analyses of ELF4, EFL1, EFL2, EFL3, and EFL4. The sequence similarity between each pair of protein is indicated. (B) Sequence alignment of ELF4, EFL1, EFL2, EFL3, and EFL4. Multiple Sequence Alignment in DNAMAN was used to align protein sequences. Full alignment and default parameters were chosen for the alignment. The phylogenetic tree was automatically produced based on the alignment.

# Supplemental Figure 5

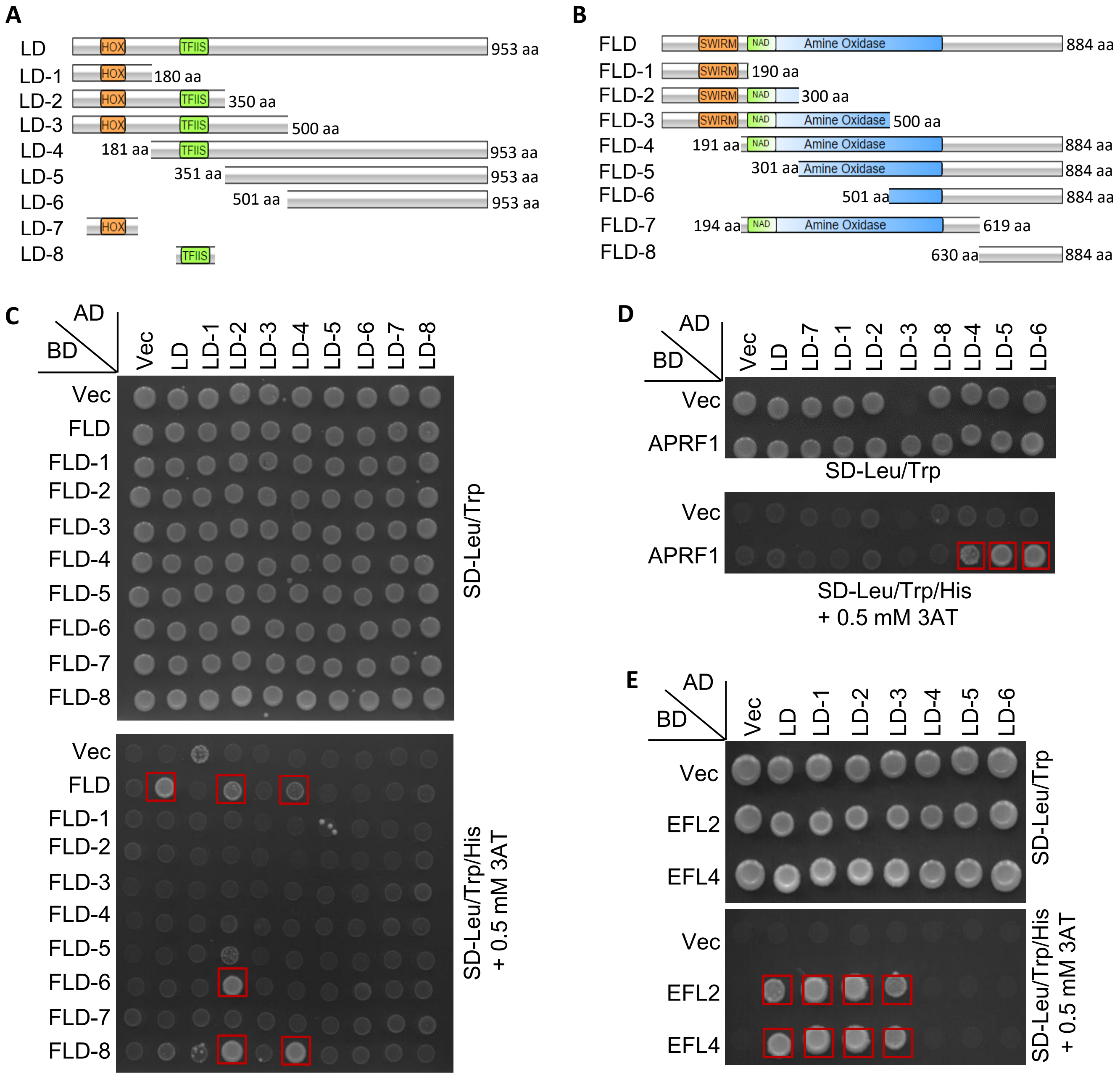


**Supplemental Figure 5. Identification of the interaction domains of FLD and LD by Y2H assays.** (A,B) Schematic representation of the full-length LD and FLD and a series of truncated versions of LD (A) and FLD (B) used in Y2H assays. The full-length and truncated versions of FLD and LD fused with GAL4-BD and/or GAL4-AD were subjected to Y2H assays. The conserved domains in LD and FLD are shown. (C) Determination of the FLD and LD domains responsible for the FLD-LD interaction by Y2H assays. The full-length and truncated versions of FLD fused with GAL4-BD and the full-length and truncated version of LD fused with GAL4-BD were co-expressed and subjected to the Y2H assays. (D) Determination of the interaction between APRF1 and a series of truncated versions of LD by Y2H assays. (E) Determination of the interaction between EFL2/4 and a series of truncated versions of LD by Y2H assays. The yeast strains harboring both the GLA4-BD and GAL4-AD plasmids were grown on the SD-Leu/Trp medium and the SD-Leu/Trp/His medium supplemented with 0.5 mM 3-AT.

# Supplemental Figure 6

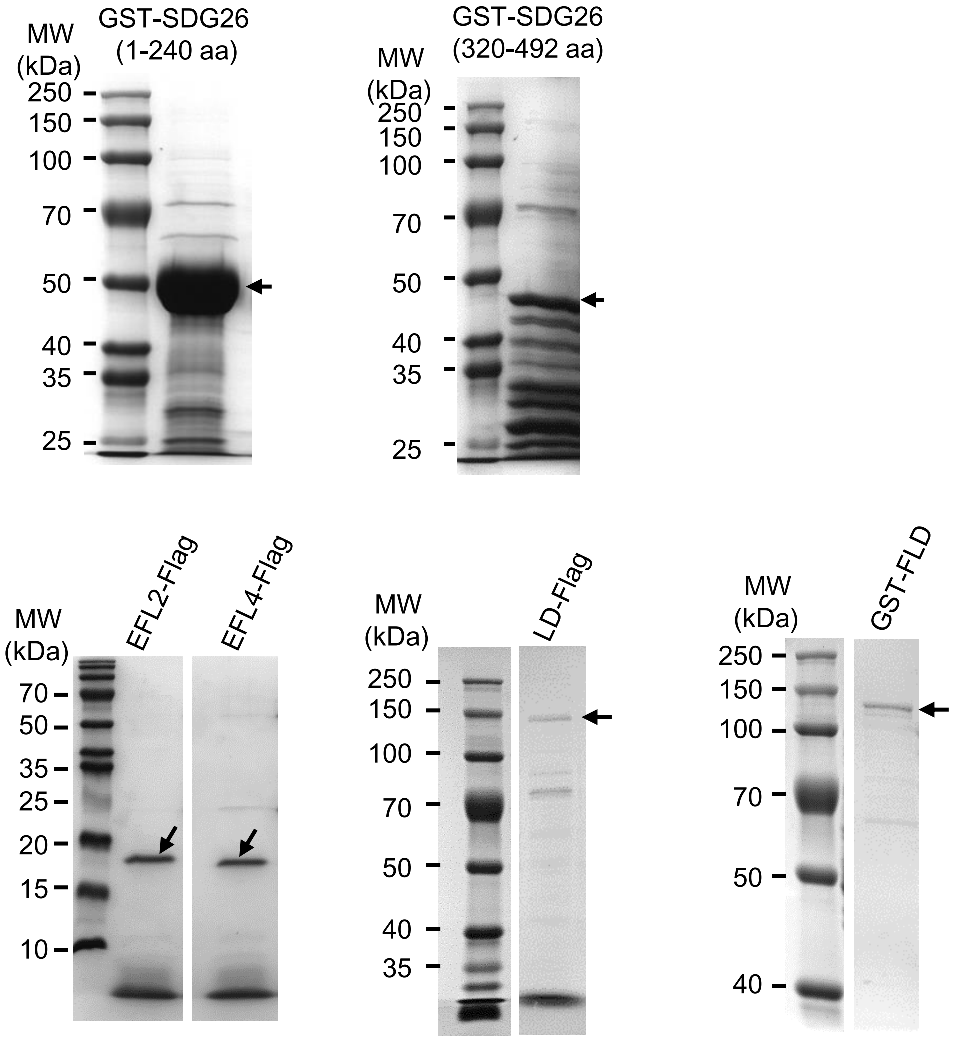


**Supplemental Figure 6. Expression and purification of tagged SDG26, EFL2, EFL4, LD, and FLD proteins.** Proteins tagged by GST were expressed and purified from *E. coli*, and the proteins tagged by Flag were expressed and purified from yeast. The purified proteins were subjected to pull-down assays and/or electrophoretic mobility shift assays. Target proteins are labelled by arrows. The molecular weight marks are shown next to the purified proteins.

# Supplemental Figure 7

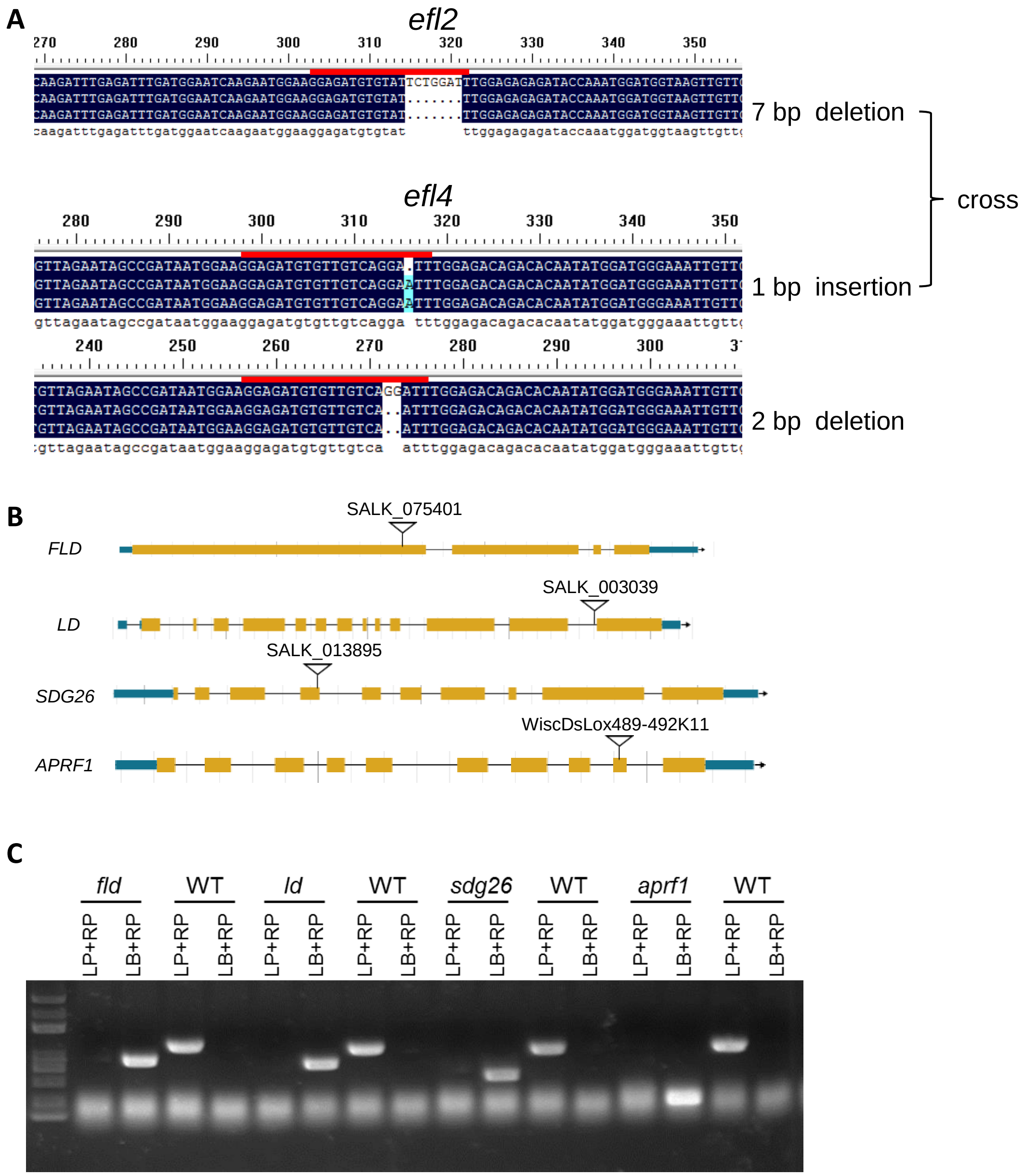


**Supplemental Figure 7. Genotying of the autonomous pathway mutants used in this study.** (A) Diagrams showing mutated *EFL2* and *EFL4* sequences aligned with their WT sequences. Red lines highlight the sequences of guide RNAs used for generating mutations by CRISPR-Cas9. *EFL2* and *EFL4* were independently mutated in the WT Col-0 plants. The 7-bp deletion *efl2* mutant was crossed with the 1-bp deletion *efl4* mutant, and the *efl2/4* double mutant was subsequently identified in the progeny. (B) *FLD*, *LD*, *SDG26* and *APRF1* genomic organization and location of T-DNA insertions. Yellow boxes, grey lines, and blue boxes represent exons, introns, and untranslated regions, respectively. Triangles indicate the position of T-DNA insertions. (C) Verification of the T-DNA insertion by amplification of genomic DNA isolated from WT plants and mutants. LB, Left border primer of the T-DNA insertion; LP, Left genomic primer; RP, Right genomic primer.

# Supplemental Figure 8

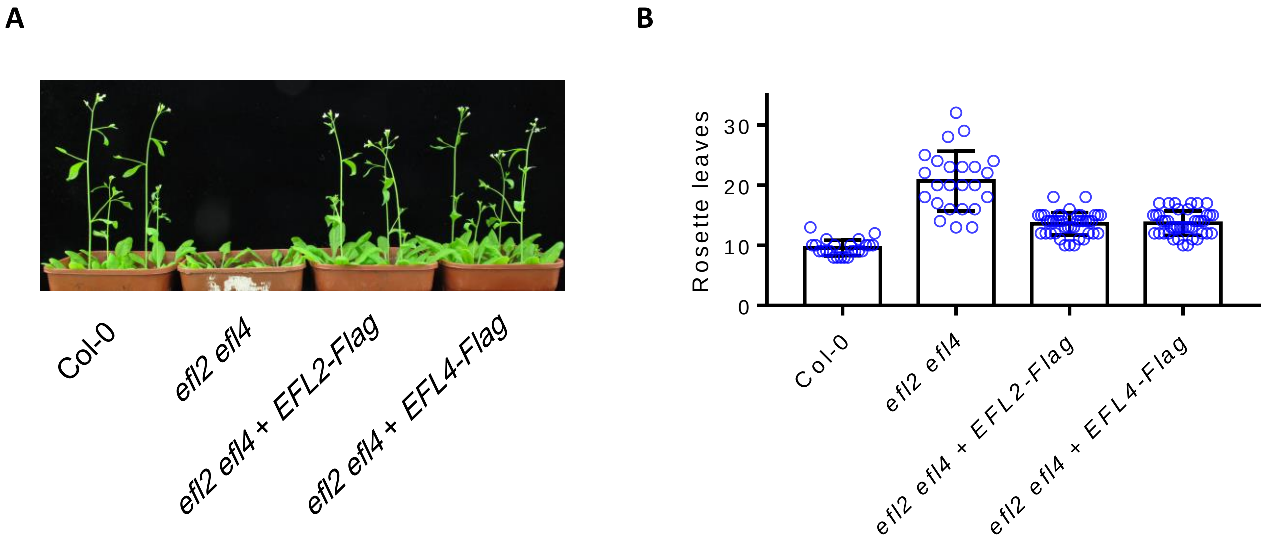


**Supplemental Figure 8. Complementation testing of the late-flowering phenotype of the *efl2 efl4* double mutant.** (A) The phenotype of WT, *efl2 efl4*, and the complementation lines harboring a native promoter-driven *EFL2* or *EFL4* tagged by a Flag epitope. Four-week-old plants grown under long days are shown. (B) The numbers of rosette leaves of bolting plants gown under long days. Values are means ± SD (n>24).

# Supplemental Figure 9

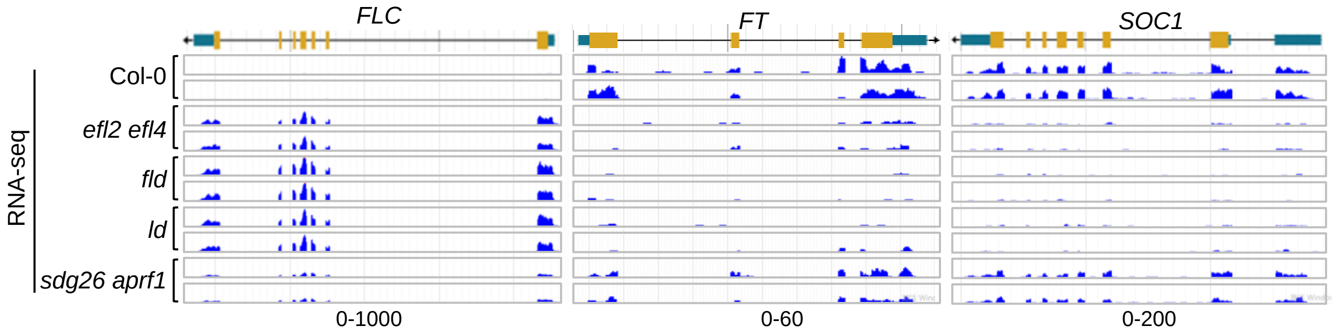


**Supplemental Figure 9. Genome browser view of RNA-seq reads at *FLC*, *FT*, and *SOC1* in the APC mutants and the WT.** The scale of RPKM is shown for each gene.

# Supplemental Figure 10

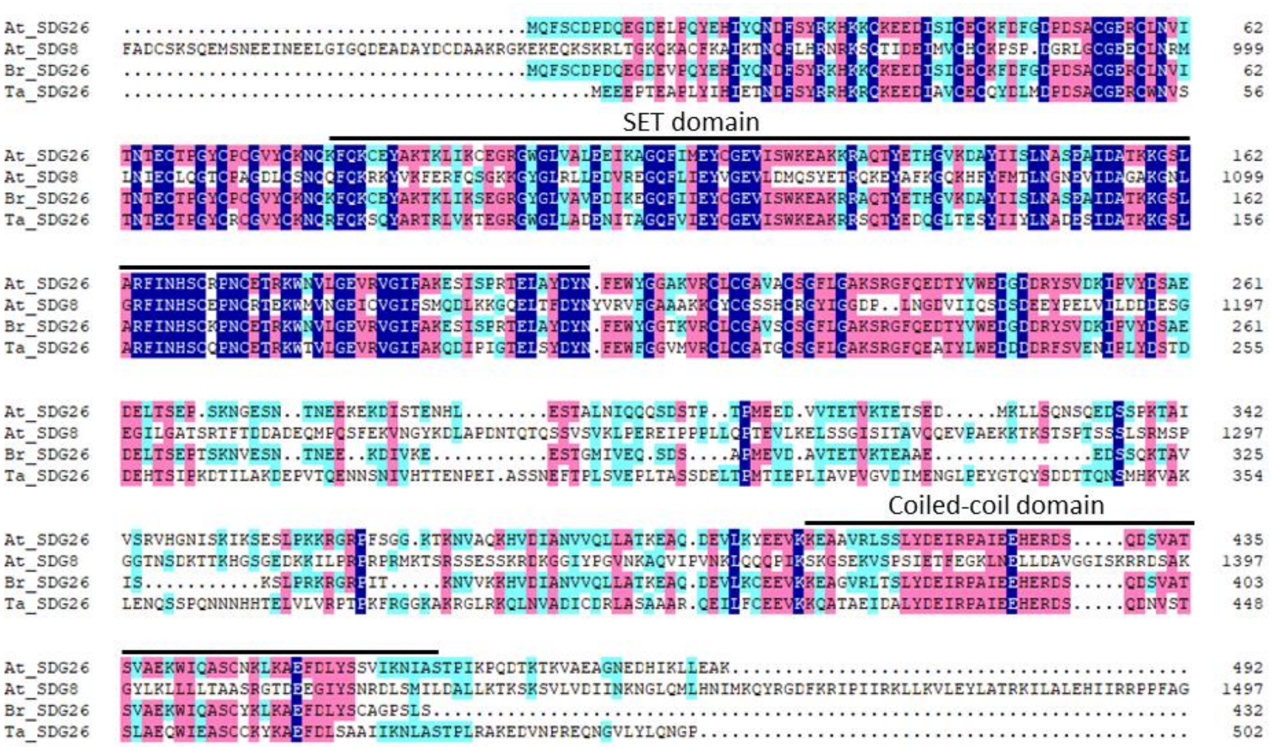


**Supplemental Figure 10. Conservation of the SET and C-terminal coiled-coil domains of the SDG26 orthologs in angiosperms.** Sequence alignments of Arabidopsis SDG26 and SDG26 orthologs in rape and wheat, and the SDG26-related histone methyltransferase SDG8 in Arabidopsis. The conserved SET and coiled-coil domains are marked.

# Supplemental Figure 11

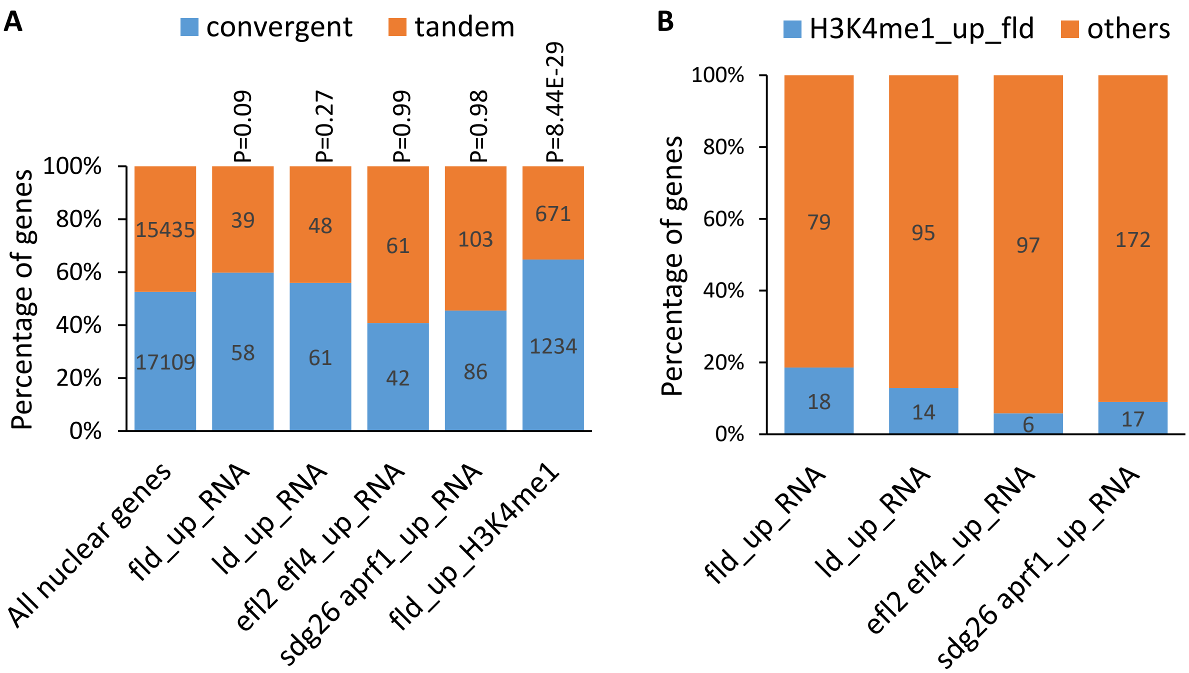


**Supplemental Figure 11. Analyses of up-regulated DEGs in the AuPC complexes.** (A) Proportions of convergent and tandem genes among genes with increased expression in the AuPC mutants and among genes with increased H3K4me1 levels (FDR<0.05, fold change>1.2) in *fld*. All the Arabidopsis nuclear genes were classified as convergent (17109) or tandem (15435) genes based on the orientation of their downstream genes. The chart indicates the proportion of convergent and tandem genes in genes with increased expression in the *fld*, *ld*, *efl2 efl4*, and *sdg26 aprf1* mutants and in genes with increased H3K4me1 levels in the *fld* mutant. P values were determined by hypergeometric test to assess whether up-regulated DEGs in the AuPC mutants and genes with increased H3K4me1 levels in the *fld* mutant are enriched in convergent genes. (B) Proportions of genes with FLD-dependent H3K4me1 demethylation among up-regulated DEGs in the AuPC mutants. The genes with increased levels of H3K4me1 (FDR<0.05, fold change>1.2) in the *fld* mutant were recognized as genes with FLD-dependent H3K4me1 demethylation.
