# Supplementary material for: Characterization of an autonomous pathway complex that promotes flowering in Arabidopsis
Source: Nucleic Acids Res. 2022 Jun 29;50(13):7380–95. doi: 10.1093/nar/gkac551 (PMC9303297; doi:10.1093/nar/gkac551)
Supplement: gkac551_Supplemental_Files [file gkac551_supplemental_files.zip › Supplemental Table 1.docx]

**Supplemental Table 1.** Overlap between DEGs and genes with affected histone modifications in the *fld* mutant.

| Histone modification  change (\|log2FC\|>0.585, P<0.01) | | Expression change | |
| --- | --- | --- | --- |
|  |  | Up-regulated  genes (97) | Down-regulated genes (91) |
| H3Ac | Up (9) | 2 (*FLC*) | 0 |
|  | Down (8) | 0 | 3 |
| H3K4me2 | Up (18) | 0 | 0 |
|  | Down (9) | 2 (*FLC*) | 2 |
| H3K4me3 | Up (13) | 5 (*FLC*) | 0 |
|  | Down (8) | 0 | 3 |
| H3K36me3 | Up (29) | 6 (*FLC*) | 0 |
|  | Down (3) | 0 | 1 |
| H3K27me3 | Up (3) | 0 | 1 |
|  | Down (14) | 3 (*FLC*) | 0 |

Note: The numbers of overlaps between differentially expressed genes and genes with affected histone modifications in the *fld* mutant are shown. *FLC* is shown in brackets when it is present in the overlapping genes.
